# Chinese herbal medicine as adjunct therapy improves clinical recovery and reduces multidrug-resistant bacterial load in older adults with pulmonary infection: a retrospective cohort study

**DOI:** 10.3389/fmed.2026.1762339

**Published:** 2026-04-28

**Authors:** Chunyan Wang, Siyu Wang, Fangxiao Hu, Anna Zhang, Qiao Yin, Shaojie Bi, Ning Wei, Qingbo Zhou

**Affiliations:** 1 Department of Geriatrics, Integrated Traditional Chinese and Western Medicine Center, The Second Qilu Hospital of Shandong University, Shandong University, Jinan, Shandong, China; 2 Department of Neurology, The Second Qilu Hospital of Shandong University, Shandong University, Jinan, Shandong, China; 3 Department of Cardiology, Integrated Traditional Chinese and Western Medicine Center, The Second Qilu Hospital of Shandong University, Shandong University, Jinan, Shandong, China; 4 Department of Information Center, The Second Qilu Hospital of Shandong University, Shandong University, Jinan, Shandong, China; 5 Geriatric Medicine Center, Affiliated Hospital of Shandong University of TCM, Jinan, Shandong, China

**Keywords:** adjunct therapy, Chinese herbal medicine, clinical recovery, multidrug-resistant bacteria, older adults, pulmonary infection

## Abstract

**Objective:**

Pulmonary infections are common and challenging in older adults due to immune decline, comorbidities, and antimicrobial resistance. This study aimed to evaluate the effects of Chinese herbal medicine as an adjunct therapy to improve immune function and infection outcomes.

**Methods:**

This retrospective cohort study was conducted at the Department of Geriatrics, the Second Hospital of Shandong University, China, and included 372 older adults who were admitted between 1 January 2024 and 30 June 2024. Patients were divided into two groups: those who received CHM treatment and those who received conventional treatment. Data were retrieved from the hospital’s information system, including patient demographics, clinical status, laboratory results, and treatment outcomes. Outcomes included hospital stay time, febrile duration, symptom recovery, SpO_2_ recovery, CPIS recovery, laboratory parameter recovery, MDR-related indicators and mortality. Propensity score matching and E-values were performed as a sensitivity analysis to address baseline confounding, with covariate balance assessed by standardized mean differences.

**Results:**

CHM treatment was associated with significantly reduced hospital stay and febrile period, as well as a higher fever resolution rate compared to conventional treatment. Inflammatory markers improved in the CHM group, suggesting a potential immunomodulatory effect. CHM-treated patients also exhibited greater reductions in MDR bacterial counts. These beneficial effects remained consistent in the PSM-matched cohort and were further validated by E-value analysis for robustness against unmeasured confounding. However, no significant differences were found in overall mortality or MDR bacterial reversal. Subgroup analysis showed greater benefits in frail, malnourished, immunocompromised, disabled, and patients with altered consciousness.

**Conclusion:**

CHM offers clinical benefits as an adjunct therapy for older adults with pulmonary infections, particularly in improving recovery time and managing MDR bacteria. Further research is required to confirm its efficacy and optimize treatment protocols.

## Introduction

According to the latest Global Burden of Disease (GBD) study data published in Lancet, lower respiratory infections remain the leading cause of mortality from infectious diseases globally. Pulmonary infection, a common type of lower respiratory infection, is characterized by high mortality rates, numerous complications, poor prognosis, and significant economic burden (1). The treatment of older adults is particularly complex due to age-related immune dysfunction, multiple chronic comorbidities, frailty, and polypharmacy, posing substantial challenges to clinical practice. Current conventional management of pulmonary infections primarily involves antimicrobial therapy, mechanical ventilation, and glucocorticoid administration. Although modern medicine has achieved remarkable progress in this field, its limitations are increasingly evident. These include antimicrobial resistance (AMR), a high proportion of multidrug-resistant (MDR) infections, delays in diagnosis and difficulties in pathogen identification, as well as suboptimal long-term efficacy, which continue to hinder effective prevention and control. These challenges have prompted growing interest in complementary and integrative medicine strategies, among which Traditional Chinese Medicine (TCM) is a notable example. The advantages of Chinese Herbal Medicine (CHM) in treating pulmonary infections are not primarily reflected in the intensity or speed of direct bactericidal action but rather in its holistic approach, multi-target regulation, and personalized treatment strategies. Consequently, CHM is gaining recognition as a potential adjunctive measure within the Infection Prevention and Control (IPC) and Antimicrobial Stewardship (AMS) frameworks for addressing infectious diseases and antimicrobial resistance.

CHM has demonstrated favorable therapeutic efficacy in the management of pulmonary infections. For instance, a multicenter, prospective, randomized controlled trial (RCT) led by Academician Zhong Nanshan’s research team evaluated the efficacy and safety of Lianhua Qingwen Capsule in treating patients with COVID-19. The results indicated that add-on therapy with Lianhua Qingwen Capsule for 14 days, based on conventional treatment, significantly improved the clinical recovery rate, shortened the time to symptom resolution, and exhibited a favorable safety profile (2). Furthermore, a meta-analysis incorporating 51 RCTs (involving 5,799 children aged 1–14 years) revealed that the combination of CHM and azithromycin for pediatric pneumonia yielded superior clinical efficacy and safety compared to antibiotic monotherapy (3). Another clinical trial observed that after a 12-week CHM intervention, the cumulative incidence of pneumonia and related hospitalization rates were significantly reduced (4). These studies collectively suggest that CHM plays a beneficial role in the prevention and treatment of pulmonary infections. Concurrently, owing to their multi-component and multi-pathway characteristics, CHM are regarded as a novel source of antimicrobial agents to combat drug-resistant bacteria. A recent systematic review and meta-analysis has further confirmed that integrated traditional Chinese and Western medicine therapy significantly enhances the clinical efficacy of multidrug-resistant bacterial pneumonia and reduces the bacterial load of drug-resistant pathogens, providing high-level evidence for CHM as an adjunctive strategy for MDR infection management (5). Zhao et al. reviewed the antimicrobial effects of bioactive compounds isolated from CHM, further supporting the potential of CHM in combating bacterial drug resistance (6). Furthermore, Feng et al. identified tanshinones, compounds from TCM, as effective inhibitors of the Type 3 Secretion System (T3SS) in *Pseudomonas aeruginosa*, a multidrug-resistant bacterium (7). Additionally, Liu et al. discussed the antimicrobial mechanisms of CHM, noting that combining CHM with antibiotics can restore antibiotic sensitivity in resistant strains (8). Notably, Academician Zhong Nanshan’s team also elaborated on the critical role of TCM in antimicrobial stewardship (AMS) from both clinical and basic research perspectives, pointing out that CHM’s immunomodulatory and anti-virulence properties can effectively reduce antibiotic use and alleviate the pressure of antimicrobial resistance (AMR), which aligns with the global AMS development goal (9). These results collectively illustrate that CHM has immunomodulatory and anti-inflammatory effects and can contribute to controlling MDR infections, further supporting its potential value in AMS.

However, there is currently a lack of systematic evaluation regarding the efficacy and safety of adjunctive CHM therapy specifically for older adults with pulmonary infections. Therefore, this study employed a retrospective cohort design to assess the association between CHM treatment and clinical outcomes in older adults with pulmonary infections. It focused on analyzing indicators relevant to IPC and AMS, including length of hospital stay (LOS), time to fever resolution, recovery of inflammatory markers, and the detection rate of multidrug-resistant organisms (MDROs). This comprehensive approach aims to evaluate the quality of evidence for adjunctive CHM therapy in this patient population, provide reliable evidence for its efficacy and safety assessment, and offer directions for clinical practice and further research.

## Methods

### Study design and population

This retrospective cohort study was conducted at the Department of Geriatrics, the Second Hospital of Shandong University, Shandong, China. A total of 372 older adults (≥65 years) diagnosed with pulmonary infections were admitted from 1 January 2024 to 30 June 2024. Patient data were retrieved from the Hospital Information System, which provided information on demographic characteristics, clinical status, laboratory test results, and treatment outcomes. The study protocol was reviewed and approved by the Research Ethics Committee of the Second Hospital of Shandong University [KYLL-2023(KJ)P-1792]. Given the retrospective design and anonymized dataset, the requirement for informed consent was formally waived. All procedures adhered to the principles of the Declaration of Helsinki.

### Inclusion and exclusion criteria

Inclusion criteria consisted of older adults (≥65 years) who were diagnosed with pulmonary infections and had complete medical records. This age threshold was based on internationally accepted definitions of older adults (10), whereas the actual age range of enrolled patients was determined by consecutive case availability during the study period, without any predefined upper age limit. Pulmonary infections were diagnosed according to the diagnostic criteria established by the World Health Organization (WHO) and the Guidelines for the Management of Adults with Community-Acquired Pneumonia (CAP) jointly published by the American Thoracic Society (ATS) and the Infectious Diseases Society of America (IDSA) (11), including clinical symptoms such as cough, fever, sputum production, and radiographic evidence of lung consolidation or infiltration. Patients were classified as mild or severe based on clinical presentation and assessment tools such as the Clinical Pulmonary Infection Score (CPIS) and oxygenation status. Exclusion criteria included (i) Patients with missing clinical data or those who were lost to follow-up during hospitalization, (ii) Patients presenting with impaired renal or hepatic function at admission were excluded from this study, (iii) Patients on long-term immunosuppressive therapy or glucocorticoids, those with a history of acquired immunodeficiency or organ transplantation, those who had undergone radiotherapy or chemotherapy for oncologic or hematologic malignancies, and those who had experienced diabetic emergencies or hospital-acquired infections were also excluded. All eligible patients were consecutively enrolled during the study period to reflect real-world clinical practice, without selective exclusion of specific baseline characteristics.

### Grouping and intervention

Patients enrolled in the study were divided into a CHM group and a Non-CHM group. The CHM group received, in addition to conventional treatment, syndrome differentiation and treatment by a licensed CHM practitioner in accordance with the *Guidelines of integrated traditional Chinese and Western medicine for diagnosis and treatment of CAP* issued by Internal Medicine Committee of World Federation of Chinese Medicine Societies (12). Patients diagnosed with the wind-heat attacking lung syndrome were prescribed Yinqiao San (YQS) and those diagnosed with the phlegm-heat obstructing the lung syndrome were prescribed Qingjin Huatan Decoction (QJHTD). The composition, dosage and diagnostic principles of QJHTD and YQS are detailed in Supplementary Table S1. QJHTD and YQS were administered in granule form, all to be taken orally twice daily mixed with 200 mL of warm water. CHM granules were initiated within 24 h of diagnosis and administered orally twice daily for 7–14 consecutive days according to syndrome differentiation. Supplementary Table S2 lists the Latin names, manufacturing companies and National Medical Insurance Code of the relevant CHM. The Non-CHM group received management per the *Guidelines for the Management of Adults with CAP*, which included general supportive care, supplemental oxygen therapy, mechanical ventilation, and appropriate antibiotic and antiviral therapy. The antibiotic type, dosage, and treatment duration were determined by attending physicians according to guidelines and microbial culture results, and were consistent between the two groups to avoid confounding by antibiotic exposure (Supplementary Table S3).

### Data collection

Patient baseline characteristics, including age, gender, body mass index (BMI), comorbidities, performance status, nutritional status, conscious state, and clinical manifestations (fever, cough and sputum, SpO_2_) were extracted from the Hospital Information System. Laboratory test results, including white blood cell (WBC) count, neutrophil (NEU) count, lymphocyte (LYM) count, C-reactive protein (CRP), procalcitonin (PCT), albumin (ALB), fibrin degradation product D (D-Dimer) and multidrug-resistant (MDR) relevant biomarkers were collected from the Department of Clinical Laboratory the Second Hospital of Shandong University. MDR bacteria were identified using standard microbiological culture and antimicrobial susceptibility testing in accordance with *Clinical and Laboratory Standards Institute (CLSI) guidelines* (13). MDR was defined as non-susceptibility to at least one agent in three or more antimicrobial categories.

### Clinical outcomes

The primary outcomes included hospital stay duration, febrile duration, and recovery from fever, cough and sputum, conscious state, SpO_2_, various laboratory parameters (WBC, NEU, LYM, CRP, PCT, ALB, D-Dimer), clinical index (CPIS, NLR, PAR) and the change of MDR status. Hospital stay was defined as the number of days from admission to discharge. Febrile duration was defined as the number of days from the first recorded fever to the first temperature <37.3 °C for at least 24 h without antipyretic use. Fever recovery was defined as defervescence maintained for ≥24 h. Symptom recovery referred to complete resolution of cough and sputum. SpO2 recovery was defined as an increase to ≥93% on room air and maintained for ≥24 h. CPIS improvement was defined as a reduction of ≥2 points from baseline. MDR bacterial count reduction was defined as a ≥ 2-log10 CFU/mL decrease or negative conversion in serial cultures. MDR reversal was defined as conversion from MDR-positive to MDR-negative in follow-up cultures. Secondary outcome was mortality. CPIS was calculated based on five parameters: body temperature, WBC count, sputum culture results, chest X-ray findings, and the oxygenation index (PaO_2_/FiO_2_ ratio) (14). Neutrophil-to-Lymphocyte Ratio (NLR) was defined as the absolute NEU count divided by the absolute LYM count, serving as a marker of systemic inflammation and immune response (15). Procalcitonin-to-Albumin Ratio (PAR) was defined as the serum PCT level divided by the serum ALB level, reflecting both systemic inflammation and nutritional status (16).

### Subgroup classification

Subgroup analyses were conducted to assess the impact of baseline characteristics on treatment outcomes. The following variables were used to define the subgroups: age (<80 years vs. ≥80 years), BMI (<18.5, 18.5–23.9, ≥24), ALB (<30 g/L vs. ≥30 g/L), disease severity (mild vs. severe), performance status (self-care, semi-disability, total disability), nutritional risk (a total score of NRS-2002 ≥ 3 indicates a clinically relevant risk of malnutrition, necessitating nutritional intervention), conscious state (awake, confused, comatose), and presence of MDR bacteria (yes vs. no). This approach allowed for examination of the differential effects of CHM treatment across various patient subgroups.

### Statistical analysis

Descriptive statistics were used to summarize baseline characteristics and clinical outcomes. Continuous variables were presented as median with interquartile range (IQR), and categorical variables were expressed as counts and percentages (%). Comparisons between the CHM and non-CHM groups were performed using the Mann–Whitney U test for continuous variables and the Chi-square test for categorical variables. Univariate and multivariate regression analyses were conducted to identify factors associated with clinical outcomes: linear regression models were used for continuous outcomes (e.g., hospital stay, febrile duration) and results were reported as *β* values with 95% confidence intervals (CIs); logistic regression models were used for binary outcomes (e.g., fever recovery, mortality) and results were reported as odds ratios (ORs) with 95% CIs.

Three multivariate models were constructed: a non-adjusted model, a minimally adjusted model (adjusted for gender and comorbidities), and a fully adjusted model (adjusted for gender, age, BMI, comorbidities, disability status, nutritional risk, conscious state, fever status, MDR infection, WBC count, CRP level, and ALB level).

To further address potential confounding from baseline imbalances and validate the robustness of our findings, propensity score matching (PSM) was performed as a sensitivity analysis (17). Propensity scores were estimated using a logistic regression model with CHM treatment as the dependent variable, and the aforementioned fully adjusted covariates as independent variables. These covariates were selected based on theoretical relevance to treatment allocation and baseline imbalance, and were confirmed to be independent of the causal pathway between CHM treatment and clinical outcomes to avoid over-matching bias. A 1:1 nearest-neighbor matching method with a caliper of 0.05 was used to match patients in the CHM and non-CHM groups. Covariate balance after matching was quantified by standardized mean differences (SMDs), with SMD < 0.1 considered indicative of adequate balance. Baseline characteristics before and after matching were compared to assess covariate balance, and visualized using propensity score distribution plots and SMD comparison plots. Clinical outcomes were re-analyzed in the matched cohort to confirm the consistency of the association between CHM treatment and key outcomes. Conditional logistic regression was used for binary outcomes and conditional linear regression for continuous outcomes in the matched cohort, accounting for the paired nature of the matched samples.

To conduct a more in-depth assessment of the impact of unmeasured confounding on key outcomes, E-values were calculated for all statistically significant associations between CHM treatment and clinical outcomes. An E-value > 1.5 was considered indicative of robust associations, meaning the observed effects are unlikely to be altered by unmeasured confounding (18).

Stratified analyses were performed to examine the association between CHM treatment and clinical outcomes in different patient subgroups. Kaplan–Meier survival curves with log-rank tests were used to assess overall survival. Survival time was defined as the number of days from hospital admission to in-hospital death. Patients discharged alive were treated as censored at the date of discharge. The maximum follow-up time corresponded to the longest hospitalization duration within the study cohort. And subgroup effects were visualized using forest plots. All hypotheses were tested at a significance level of 0.05. Analyses were performed with the statistical software packages R (The R Foundation)^1^ and Free Statistics software version 2.1.

## Results

### Baseline characteristics

A total of 372 older hospitalized patients with pulmonary infections from the Second Hospital of Shandong University were included in this study, with 145 patients receiving CHM treatment and 227 patients receiving conventional treatment alone (Table 1). The median age of the total cohort was 81.0 years (IQR: 74.0, 86.0), with the CHM group being significantly older than the non-CHM group (83.0 vs. 79.0 years, *p* < 0.001). The median BMI for all patients was 20.2 (IQR: 17.6, 21.0), with a significantly lower BMI in the CHM group (18.3 vs. 20.2, *p* < 0.001). Hypertension was present in 61.8% of the total cohort, with a significantly lower prevalence in the CHM group (52.4% vs. 67.8%, *p* = 0.003). No significant differences were observed in the prevalence of cardiovascular disease (71.7% vs. 74.0%, *p* = 0.628) or diabetes (35.2% vs. 40.5%, *p* = 0.300).

**Table 1:** Baseline characteristics of older adults with pulmonary infection before treatment.

Variables	Total (*n* = 372)	CHM group (*n* = 145)	Non-CHM group (*n* = 227)	*p*-value
Age (years), Median (IQR)	81.0 (74.0, 86.0)	83.0 (78.0, 88.0)	79.0 (73.0, 85.0)	< 0.001
Gender (male), *n* (%)	165 (44.4)	56 (38.6)	109 (48)	0.075
BMI, Median (IQR)	20.2 (17.6, 21.0)	18.3 (16.4, 20.2)	20.2 (19.7, 22.7)	< 0.001
Comorbidities, *n* (%)				
Hypertension	230 (61.8)	76 (52.4)	154 (67.8)	0.003
Cardiovascular disease	272 (73.1)	104 (71.7)	168 (74)	0.628
Diabetes	143 (38.4)	51 (35.2)	92 (40.5)	0.3
Cerebrovascular disease	205 (55.1)	86 (59.3)	119 (52.4)	0.193
Severity, *n* (%)	50 (13.4)	20 (13.8)	30 (13.2)	0.874
CPIS score, Median (IQR)	5.4 (5.0, 6.0)	5.4 (5.0, 6.0)	5.4 (5.0, 5.4)	0.303
Performance status, *n* (%)				< 0.001
Self-care	180 (48.4)	11 (7.6)	169 (74.4)	
Semi-disability	28 (7.5)	12 (8.3)	16 (7)	
Total disability	164 (44.1)	122 (84.1)	42 (18.5)	
Nutritional risk, *n* (%)				< 0.001
Yes	200 (53.8)	121 (83.4)	79 (34.8)	
No	172 (46.2)	24 (16.6)	148 (65.2)	
Conscious status, *n* (%)				< 0.001
Awake	248 (66.7)	73 (50.3)	175 (77.1)	
Confused	105 (28.2)	61 (42.1)	44 (19.4)	
Comatose	19 (5.1)	11 (7.6)	8 (3.5)	
Fever, *n* (%)	136 (36.6)	125 (86.2)	111 (48.9)	< 0.001
Temperature Max (°C), Median (IQR)	37.6 (36.9, 38.3)	38.0 (37.2, 38.5)	37.3 (36.8, 38.1)	< 0.001
Cough and sputum, *n* (%)	307 (82.5)	128 (88.3)	179 (78.9)	0.02
SpO_2_ (%), Median (IQR)	96.0 (94.6, 97.0)	96.0 (94.6, 97.0)	95.0 (94.6, 97.0)	0.088
MDR, *n* (%)	129 (34.7)	76 (52.4)	53 (23.3)	< 0.001
Laboratory tests, Median (IQR)				
WBC (×10^9^/L)	7.47 (5.69, 9.83)	8.61 (6.33, 11.53)	6.80 (5.29, 9.23)	< 0.001
NEU (×10^9^/L)	6.60 (4.34, 9.41)	6.69 (4.57, 9.49)	6.53 (4.21, 9.24)	0.495
LYM (×10^9^/L)	1.11 (0.75, 1.54)	1.14 (0.77, 1.56)	1.11 (0.71, 1.54)	0.579
NLR	6.18 (3.14, 11.69)	6.01 (3.24, 11.05)	6.28 (3.12, 11.92)	0.997
CRP (mg/L)	34.42 (8.68, 92.62)	42.34 (13.38, 101.64)	28.09 (6.56, 83.90)	0.012
PCT (ng/mL)	0.13 (0.05, 0.46)	0.14 (0.06, 0.53)	0.12 (0.05, 0.33)	0.587
ALB (g/L)	34.0 (30.4, 37.8)	31.4 (27.8, 34.4)	35.3 (33.0, 39.5)	< 0.001
PAR	0.0044 (0.0017, 0.0148)	0.0044 (0.0019, 0.0208)	0.0040 (0.0015, 0.0130)	0.254
D-Dimer (ug/mL)	1.43 (0.88, 2.52)	1.85 (1.24, 3.37)	1.20 (0.68, 1.95)	< 0.001

The inclusion criterion for age was ≥65 years, in accordance with the internationally accepted definition for older adults in geriatric research. The age range presented in this table reflects the actual distribution of consecutively enrolled eligible patients, with no predefined upper age limit applied. The minor difference in maximum age between the study groups is attributable to real-world clinical case availability, and age-related confounding has been effectively adjusted for using PSM, as detailed in Supplementary Table S3.

CHM, Chinese herbal medicine; IQR, interquartile range; BMI, body mass index; CPIS, clinical pulmonary infection score; SpO_2_, peripheral capillary oxygen saturation; MDR, multidrug resistant; WBC, white blood cells; NEU, neutrophil; LYM, lymphocyte; NLR, neutrophil-to-lymphocyte ratio; CRP, C-reactive protein; PCT, procalcitonin; ALB, albumin; PAR, procalcitonin-to-albumin ratio; D-Dimer, fibrin degradation product D; PSM, propensity score matching.

The CHM group exhibited a significantly higher proportion of patients with total disability (84.1% vs. 18.5%, *p* < 0.001) and nutritional risk (83.4% vs. 34.8%, *p* < 0.001). Conscious status differed significantly between groups, with fewer patients in the CHM group being awake (50.3% vs. 77.1%, *p* < 0.001) and a higher proportion being confused (42.1% vs. 19.4%, *p* < 0.001). Fever was more prevalent in the CHM group (86.2% vs. 48.9%, *p* < 0.001), with a higher maximum temperature (38.0 °C vs. 37.3 °C, *p* < 0.001). Cough and sputum symptoms were reported in 82.5% of the total cohort, with a higher prevalence in the CHM group compared to the non-CHM group (88.3% vs. 78.9%, *p* = 0.020). The CHM group had a significantly higher prevalence of MDR bacterial infections (52.4% vs. 23.3%, *p* < 0.001).

Regarding laboratory parameters, the CHM group had significantly higher median WBC counts (8.61 vs. 6.80 × 10 (9)/L, *p* < 0.001) and CRP levels (42.34 vs. 28.09 mg/L, *p* = 0.012), but significantly lower ALB levels (31.4 vs. 35.3 g/L, *p* < 0.001). No significant differences were observed between the groups in severity classification (*p* = 0.874), CPIS score (*p* = 0.303), or SpO_2_ levels (*p* = 0.088) at baseline.

To mitigate the impact of baseline imbalances, 1:1 PSM was performed, resulting in 132 matched pairs (CHM group *n* = 132, non-CHM group *n* = 132). After matching, all baseline covariates were well-balanced between the two groups (all *p* > 0.05, Supplementary Table S3), including key factors such as age (82.0 vs. 81.0 years, *p* = 0.352), BMI (18.5 vs. 18.7, *p* = 0.486), MDR infection (51.5% vs. 50.0%, *p* = 0.783), WBC count (8.42 vs. 8.25 × 10 (9)/L, *p* = 0.574), CRP level (40.12 vs. 38.56 mg/L, *p* = 0.618), and ALB level (31.7 vs. 32.0 g/L, *p* = 0.532), confirming effective elimination of confounding bias. Covariate balance was further visualized by propensity score distribution plots and SMD analysis (Supplementary Figures S1, S2), with SMD values <0.1 for most covariates after matching. The propensity score model showed good goodness-of-fit (C-statistic = 0.78, 95% CI: 0.73–0.83; Hosmer-Lemeshow test *p* = 0.36), indicating reliable prediction of CHM treatment allocation.

### Differences in clinical outcomes between CHM and non-CHM groups

Table 2 presents the primary and secondary clinical outcomes comparing the CHM and non-CHM groups. The primary outcomes included hospital stay time, febrile duration, symptom recovery (fever, cough and sputum, consciousness), SpO_2_ recovery, CPIS recovery, laboratory parameter recovery (WBC, NLR, CRP, PCT, PAR, D-Dimer), and MDR-related indicators. While the secondary outcome was mortality.

**Table 2:** Clinical outcomes between the Chinese herbal medicine and non-herbal medicine groups.

Outcomes	Total (*n* = 372)	CHM group (*n* = 145)	Non-CHM group (*n* = 227)	*p*-value
Primary outcome				
Hospital stay (days), Median (IQR)	18.0 (14.0, 22.3)	16.0 (9.0, 21.0)	18.0 (18.0, 22.9)	< 0.001
Febrile duration (days), Median (IQR)	3.0 (1.0, 5.0)	2.0 (1.0, 5.0)	4.0 (2.0, 5.0)	< 0.001
Fever recovery, *n* (%)	236 (63.4)	126 (86.9)	110 (48.5)	< 0.001
Cough and sputum recovery, *n* (%)	235 (63.2)	84 (57.9)	151 (66.5)	0.094
Conscious status recovery, *n* (%)	47 (12.6)	27 (18.6)	20 (8.8)	0.005
SpO_2_ Recovery, *n* (%)	233 (62.6)	92 (63.4)	141 (62.1)	0.795
CPIS score recovery, *n* (%)	130 (34.9)	58 (40)	72 (31.7)	0.102
WBC recovery, *n* (%)	88 (23.7)	44 (30.3)	44 (19.4)	0.015
NLR recovery, *n* (%)	226 (60.8)	90 (62.1)	136 (59.9)	0.678
CRP recovery, *n* (%)	154 (41.4)	84 (57.9)	70 (30.8)	< 0.001
PCT recovery, *n* (%)	106 (28.5)	47 (32.4)	52 (22.9)	0.047
PAR recovery, *n* (%)	76 (20.4)	33 (22.8)	43 (18.9)	0.373
D-Dimer recovery, *n* (%)	87 (23.4)	39 (26.9)	48 (21.1)	0.201
MDR reversal, *n* (%)	4 (1.1)	2 (1.4)	2 (0.9)	0.645
MDR bacteria number reduction, *n* (%)	82 (22.0)	61 (42.1)	21 (9.3)	< 0.001
Secondary outcome				
Mortality, *n* (%)	25 (6.7)	13 (9)	12 (5.3)	0.167

CHM, Chinese herbal medicine; IQR, interquartile range; SpO_2_, peripheral capillary oxygen saturation; CPIS, clinical pulmonary infection score; WBC, white blood cells; NLR, neutrophil-to-lymphocyte ratio; CRP, C-reactive protein; PCT, procalcitonin; PAR, procalcitonin-to-albumin ratio; D-Dimer, fibrin degradation product D; MDR, multidrug resistant.

Patients in the CHM group had a significantly shorter hospital stay compared to the non-CHM group (16 vs. 18 days, *p* < 0.001). Similarly, the febrile duration was reduced in the CHM group (2 vs. 4 days, *p* < 0.001). Fever recovery rates were significantly higher in the CHM group (86.9% vs. 48.5%, *p* < 0.001). Conscious state recovery was more prevalent in the CHM group (18.6% vs. 8.8%, *p* = 0.005). Recovery of WBC counts (30.3% vs. 19.4%, *p* = 0.015) and CRP levels (57.9% vs. 30.8%, *p* < 0.001) was also more frequently observed in CHM-treated patients.

Although MDR reversal did not show a statistically significant difference (*p* = 0.645), the reduction in MDR bacterial count was significantly greater in the CHM group (42.1% vs. 9.3%, *p* < 0.001). However, no statistically significant differences were observed in cough and sputum recovery (*p* = 0.094), SpO_2_ recovery (*p* = 0.795), CPIS score improvement (*p* = 0.102), NLR recovery (*p* = 0.678), PAR recovery (*p* = 0.373) or D-Dimer (*p* = 0.201) between the two groups. For the secondary outcome, the mortality rate was 9.0% in the CHM group and 5.3% in the non-CHM group, with a numerically higher rate in the CHM group but no statistically significant difference between the two groups (*p* = 0.167).

In the PSM-matched cohort (Supplementary Table S4), the beneficial effects of CHM treatment remained consistent and statistically significant. Accounting for the paired nature of matched samples, conditional linear regression was used for continuous outcomes and conditional logistic regression for binary outcomes: hospital stay was reduced by 3.12 days (*β* = −3.12, 95% CI: −4.58 to −1.66, *p* < 0.001), febrile duration was shortened by 1.09 days (*β* = −1.09, 95% CI: −1.67 to −0.51, *p* < 0.001), and the likelihood of MDR bacterial count reduction was significantly increased (OR = 2.25, 95% CI: 1.31 to 3.87, *p* = 0.003). These results validated the robustness of our primary findings, confirming that the observed clinical benefits of CHM were not driven by baseline confounding factors.

### Relationship between CHM and patient prognosis

Univariable and multivariable logistic regression analyses (Table 3) demonstrated a significant correlation between CHM treatment and clinical outcomes. Hospital stay, febrile duration, fever recovery, conscious state recovery, WBC, CRP, PCT, and MDR reduction were found to be significantly correlated with CHM treatment in the unadjusted Model 1, the minimally adjusted Model 2 (adjusted for gender and comorbidities), and the fully adjusted Model 3 (adjusted for gender, age, comorbidities, BMI, disability status, nutritional risk, conscious state, fever status, MDR infection, WBC count, CRP level, and ALB level).

**Table 3:** Multivariate analysis of the association between clinical outcomes and dialectical application of Chinese herbal medicine.

Outcomes	Non-adjusted model		Minimally-adjusted model		Fully-adjusted model	
	*β*/OR (95% CI)	*p*-value	*β*/OR (95% CI)	*p*-value	*β*/OR (95% CI)	*p*-value
Primary outcome						
Hospital stay (days)	−3.6 (−5.04 ~ −2.15)	<0.001	−3.37 (−4.84 ~ −1.91)	<0.001	−3.29 (−4.78 ~ −1.80)	<0.001
Febrile duration (days)	−1.16 (−1.72 ~ −0.6)	<0.001	−1.17 (−1.75 ~ −0.59)	<0.001	−1.12 (−1.70 ~ −0.54)	<0.001
Fever recovery	5.26 (3.14 ~ 8.81)	<0.001	5.24 (3.09 ~ 8.88)	<0.001	5.18 (3.01 ~ 8.89)	<0.001
Cough and sputum recovery	1.38 (0.89 ~ 2.11)	0.146	1.38 (0.89 ~ 2.16)	0.151	1.35 (0.87 ~ 2.10)	0.189
Conscious status recovery	1.57 (1.07 ~ 2)	0.006	1.54 (1.04 ~ 1.97)	0.009	1.51 (1.02 ~ 1.93)	0.012
SpO_2_ recovery	1.11 (0.72 ~ 1.71)	0.632	1.07 (0.68 ~ 1.66)	0.78	1.05 (0.67 ~ 1.63)	0.827
CPIS score improvement	1.18 (0.76 ~ 1.82)	0.103	1.18 (0.76 ~ 1.85)	0.134	1.16 (0.75 ~ 1.83)	0.156
WBC recovery	1.64 (1.04 ~ 1.7)	0.016	1.59 (1.06 ~ 1.79)	0.04	1.57 (1.04 ~ 1.76)	0.045
NLR recovery	1.04 (0.68 ~ 1.6)	0.678	1.03 (0.67 ~ 1.6)	0.62	1.02 (0.66 ~ 1.58)	0.923
CRP recovery	1.75 (1.2 ~ 1.83)	<0.001	1.84 (1.3 ~ 2)	<0.001	1.81 (1.28 ~ 1.98)	<0.001
PCT recovery	1.61 (1.05 ~ 2.59)	0.051	1.68 (1.03 ~ 2.75)	0.037	1.65 (1.01 ~ 2.70)	0.043
PAR recovery	1.44 (0.87 ~ 2.4)	0.158	1.39 (0.83 ~ 2.34)	0.212	1.37 (0.82 ~ 2.29)	0.234
D-Dimer recovery	0.73 (0.45 ~ 1.18)	0.202	0.8 (0.49 ~ 1.32)	0.39	0.78 (0.48 ~ 1.27)	0.316
MDR reversal	1.64 (0.09 ~ 4.56)	0.571	1.82 (0.1 ~ 6.92)	0.835	1.79 (0.10 ~ 6.75)	0.819
MDR bacteria number reduction	2.31 (1.4 ~ 3.8)	0.001	2.38 (1.4 ~ 4.05)	0.001	2.32 (1.35 ~ 4.00)	0.002
Secondary outcome						
Mortality	0.78 (0.58 ~ 3.98)	0.172	0.71 (0.55 ~ 3.93)	0.204	0.69 (0.52 ~ 3.85)	0.241

Non-adjusted model: no covariates were adjusted; Minimally-adjusted model: adjusted for gender and comorbidities; Fully-adjusted model: adjusted for gender, age, BMI, comorbidities, disability status, nutritional risk, conscious state, fever status, MDR infection, WBC count, CRP level, and ALB level.

OR, odds ratio; CI, confidence interval; SpO_2_, peripheral capillary oxygen saturation; CPIS, clinical pulmonary infection score; WBC, white blood cells; NLR, neutrophil-to-lymphocyte ratio; CRP, C-reactive protein; PCT, procalcitonin; PAR, procalcitonin-to-albumin ratio; D-Dimer, fibrin degradation product D; MDR, multidrug resistant.

In the fully adjusted model, CHM treatment was associated with a 3.29-day reduction in hospital stay (*β* = −3.29, 95% CI: −4.78 to −1.80, *p* < 0.001) and a 1.12-day reduction in febrile duration (*β* = −1.12, 95% CI: −1.70 to −0.54, *p* < 0.001). Fever recovery (OR = 5.18, 95% CI: 3.01–8.89, *p* < 0.001) and consciousness recovery (OR = 1.51, 95% CI: 1.02–1.93, *p* = 0.012) were also significantly improved. CHM was further associated with recovery of WBC (OR = 1.57, 95% CI: 1.04–1.76, *p* = 0.045), CRP (OR = 1.81, 95% CI: 1.28–1.98, *p* < 0.001), and PCT (OR = 1.65, 95% CI: 1.01–2.70, *p* = 0.043), indicating an anti-inflammatory effect. Additionally, CHM treatment increased the likelihood of MDR bacterial count reduction (OR = 2.32, 95% CI: 1.35–4.00, *p* = 0.002), supporting its potential role in infection control.

Sensitivity analysis using E-values further confirmed the robustness of key findings regarding the association between CHM treatment and clinical outcomes. All E-values for statistically significant associations exceeded the predefined threshold of 1.5, indicating that unmeasured confounding is unlikely to alter the observed beneficial effects in clinical practice. Detailed E-value results are presented in Supplementary Table S5.

**Figure 1:**
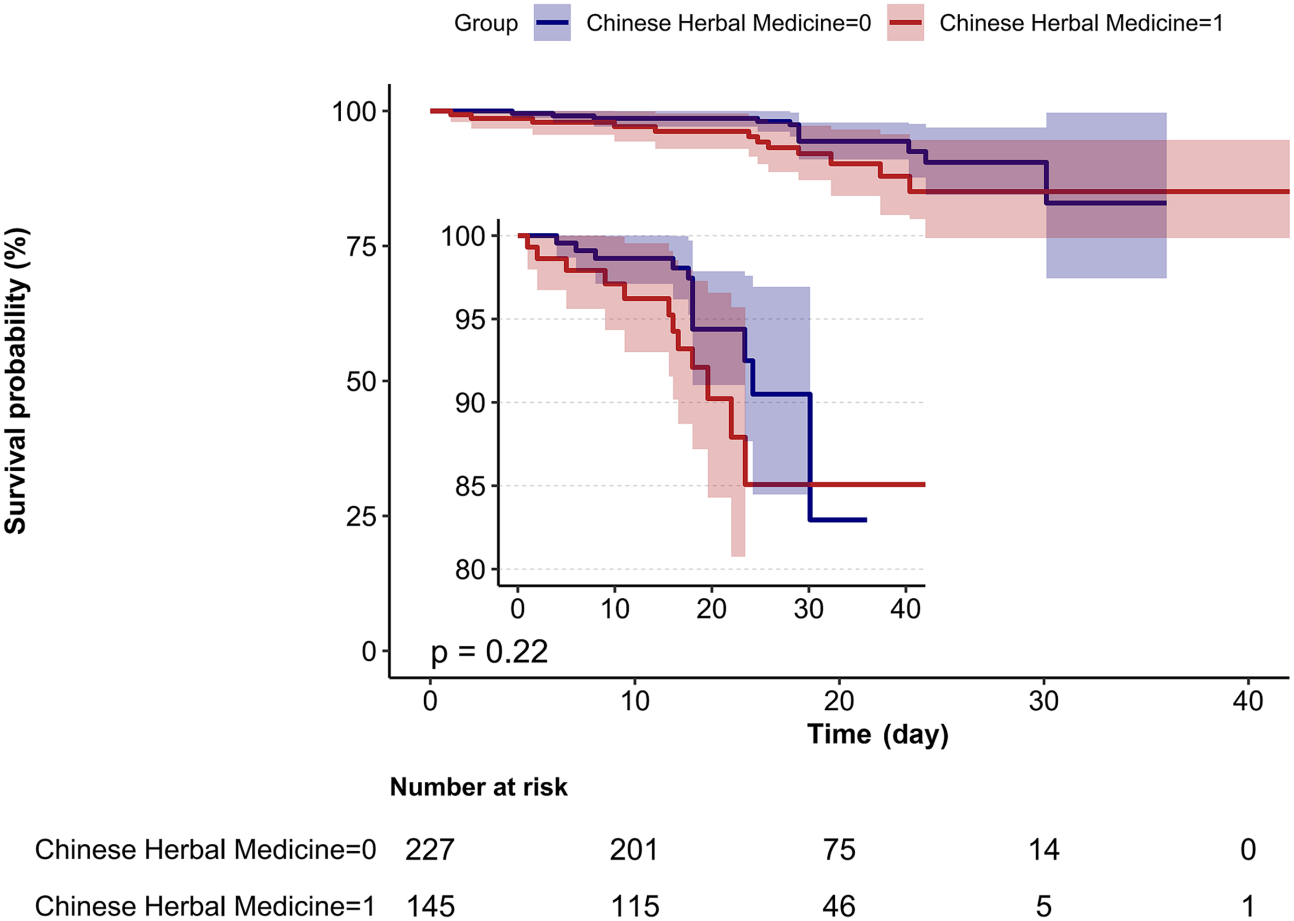
Kaplan–Meier curve for in-hospital survival (Days) of older adults with pulmonary infection between the CHM and non-CHM groups. Kaplan–Meier curves comparing in-hospital survival between the Chinese Herbal Medicine (CHM) group (red line, CHM = 1) and non-CHM group (blue line, CHM = 0). Shaded areas represent 95% CI. Survival time was defined as days from admission to in-hospital death; discharged patients were censored. Log-rank test: *p* = 0.22. Number at risk is shown below the curve. No significant difference in in-hospital survival was observed between groups.

However, CHM treatment showed no statistically significant associations with cough and sputum recovery, SpO_2_, CPIS score, NLR, PAR, D-dimer, MDR reversal, or mortality across all models. The comparison of in-hospital survival between groups, analyzed using Kaplan–Meier curves and the log-rank test, showed no statistically significant difference (Figure 1). Given the absence of significant associations in several outcomes, including in-hospital survival, we further conducted stratified analyses to evaluate the robustness of the observed significant findings and to explore potential subgroup effects in outcomes that were not significant in the overall analysis.

### Stratified analysis between CHM and patient prognosis

**Figure 2:**
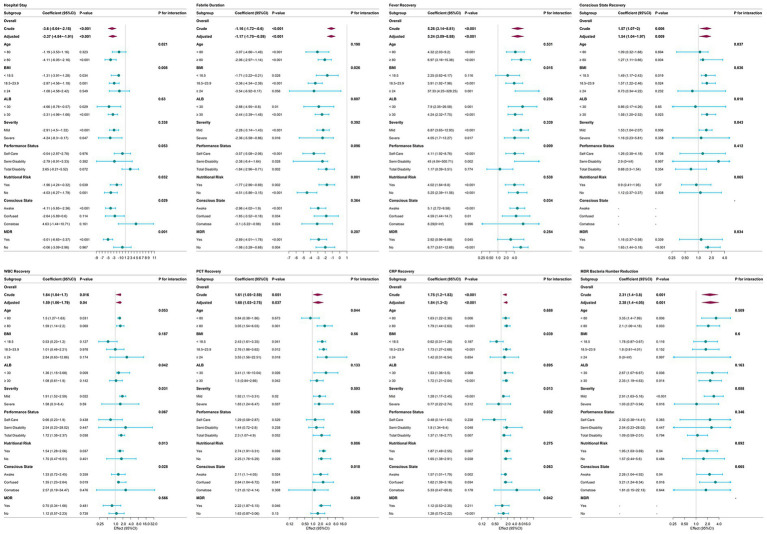
Forest plot of the main stratified analysis results. Forest plots showing subgroup analyses of the association between Chinese Herbal Medicine (CHM) treatment and key clinical outcomes in older adults with pulmonary infection. Each panel presents adjusted effect estimates (*β*/OR) with 95%CI for overall and subgroup-specific effects, alongside *p*-values for interaction tests across subgroups. Subgroups were defined by age, BMI, serum albumin level, disease severity, performance status, nutritional risk, conscious state, and multidrug-resistant (MDR) bacterial infection status. Most subgroups showed consistent beneficial effects of CHM, while significant interaction effects were observed in specific high-risk populations, highlighting the targeted value of CHM in vulnerable elderly patients.

Table 4 presents a stratified analysis of the application and clinical efficacy of CHM. Subgroup analyses demonstrated that the beneficial effects of CHM on key clinical outcomes (Figure 2)—including hospital stay, febrile duration, fever recovery, conscious status recovery, WBC, CRP and PCT recovery, as well as MDR bacterial count reduction—remained consistent across most subgroups, indicating the robustness of these findings.

**Table 4:** Stratified analysis of the association between clinical outcomes and dialectical application of Chinese herbal medicine.

Subgroup	Hospital stay			Febrile duration			Fever recovery		
	*β* (95%CI)	*p*-value	*p* for interaction	*β* (95%CI)	*p*-value	*p* for interaction	OR (95%CI)	*p*-value	*p* for interaction
Age (years)			0.021			0.198			0.531
< 80	−1.19 (−3.53 ~ 1.16)	0.323		−3.07 (−4.66 ~ −1.49)	<0.001		4.32 (2.03 ~ 9.2)	<0.001	
≥80	−4.11 (−6.05 ~ −2.16)	<0.001		−2.06 (−2.97 ~ −1.14)	<0.001		6.97 (3.16 ~ 15.38)	<0.001	
BMI			0.008			0.026			0.015
< 18.5	−1.31 (−3.91 ~ −1.28)	0.034		−1.71 (−3.22 ~ −0.21)	0.028		2.25 (0.82 ~ 6.17)	0.116	
18.5 ~ 23.9	−2.87 (−4.56 ~ −1.18)	0.001		−3.36 (−4.34 ~ −2.39)	<0.001		3.91 (1.92 ~ 7.96)	<0.001	
≥24	−1.08 (−4.58 ~ 2.42)	0.549		−3.54 (−6.92 ~ 0.17)	0.056		37.33 (4.25 ~ 328.25)	0.001	
ALB (g/L)			0.630			0.807			0.236
< 30	−4.66 (−8.76 ~ −0.57)	0.029		−2.88 (−4.95 ~ −0.8)	0.01		7.9 (2.35 ~ 26.58)	0.001	
≥30	−3.31 (−4.96 ~ −1.66)	<0.001		−2.44 (−3.39 ~ −1.48)	<0.001		4.24 (2.32 ~ 7.75)	<0.001	
Severity			0.338			0.392			0.339
Mild	−2.91 (−4.5 ~ −1.32)	<0.001		−2.28 (−3.14 ~ −1.43)	<0.001		6.87 (3.65 ~ 12.93)	<0.001	
Severe	−4.24 (−8.3 ~ −0.17)	0.047		−2.36 (−5.58 ~ −0.86)	0.016		4.05 (1.7 ~ 13.27)	0.017	
Performance status			0.053			0.096			0.009
Self-care	−0.04 (−2.87 ~ 2.78)	0.976		−3.57 (−5.08 ~ −2.06)	<0.001		4.11 (1.92 ~ 8.76)	<0.001	
Semi-disability	−2.79 (−8.91 ~ 3.33)	0.382		−2.38 (−6.4 ~ −1.64)	0.028		45 (4.04 ~ 500.71)	0.002	
Total disability	2.65 (−0.21 ~ 5.52)	0.072		−1.84 (−2.96 ~ −0.71)	0.002		1.17 (0.39 ~ 3.51)	0.774	
Nutritional risk			0.032			0.001			0.538
Yes	−1.96 (−4.24 ~ −0.32)	0.039		−1.77 (−2.86 ~ −0.69)	0.002		4.02 (1.84 ~ 8.8)	<0.001	
No	−4.03 (−6.27 ~ −1.79)	0.001		−4.51 (−5.86 ~ −3.15)	<0.001		5.25 (2.39 ~ 11.56)	<0.001	
Conscious status			0.029			0.364			0.034
Awake	−4.11 (−5.85 ~ −2.36)	<0.001		−2.96 (−4.02 ~ −1.9)	<0.001		5.1 (2.72 ~ 9.58)	<0.001	
Confused	−2.64 (−5.89 ~ 0.6)	0.114		−1.85 (−3.52 ~ −0.18)	0.034		4.59 (1.44 ~ 14.7)	0.01	
Comatose	4.63 (−1.44 ~ 10.71)	0.161		−3.1 (−5.22 ~ −0.98)	0.024		8.29 (0 ~ Inf)	0.996	
MDR			0.001			0.207			0.254
Yes	−5.01 (−6.65 ~ −3.37)	<0.001		−2.89 (−4.01 ~ −1.78)	<0.001		2.92 (0.96 ~ 8.88)	0.045	
No	−0.06 (−3.09 ~ 2.96)	0.967		−1.98 (−3.28 ~ −0.68)	0.004		6.77 (3.61 ~ 12.68)	<0.001	

Subgroup	Cough and sputum recovery			Conscious state recovery			SpO_2_ recovery		
	OR (95%CI)	*p*-value	*p* for interaction	OR (95%CI)	*p*-value	*p* for interaction	OR (95%CI)	*p*-value	*p* for interaction
Age (years)			0.030			0.037			0.688
< 80	0.92 (0.46 ~ 1.83)	0.816		1.09 (0.32 ~ 1.68)	0.894		1.17 (0.6 ~ 2.26)	0.644	
≥80	1.78 (1.02 ~ 3.12)	0.044		1.27 (1.11 ~ 3.66)	0.004		1.06 (0.6 ~ 1.88)	0.842	
BMI			0.806			0.036			0.423
< 18.5	1.53 (0.7 ~ 3.32)	0.282		1.49 (1.17 ~ 2.43)	0.019		0.69 (0.33 ~ 1.47)	0.341	
18.5 ~ 23.9	1.2 (0.62 ~ 2.33)	0.589		1.57 (1.22 ~ 2.46)	0.024		1.54 (0.79 ~ 3)	0.207	
≥24	1.4 (0.34 ~ 5.74)	0.638		0.73 (0.34 ~ 4.22)	0.232		1.22 (0.31 ~ 4.74)	0.775	
ALB (g/L)			0.501			0.018			0.663
< 30	1.72 (0.68 ~ 4.38)	0.255		0.86 (0.17 ~ 4.26)	0.85		1.19 (0.46 ~ 3.1)	0.717	
≥30	1.27 (0.74 ~ 2.15)	0.384		1.58 (1.20 ~ 2.52)	0.023		0.88 (0.52 ~ 1.49)	0.631	
Severity			0.360			0.043			0.164
Mild	1.41 (0.86 ~ 2.3)	0.174		1.53 (1.04 ~ 2.07)	0.006		1.21 (0.73 ~ 2)	0.453	
Severe	0.69 (0.22 ~ 2.14)	0.523		1.16 (0.23 ~ 5.81)	0.358		1.24 (0.29 ~ 5.38)	0.673	
Performance status			0.403			0.412			0.046
Self-care	1.15 (0.53 ~ 2.53)	0.519		1.26 (0.38 ~ 4.18)	0.708		1.32 (0.62 ~ 2.81)	0.478	
Semi-disability	0.9 (0.09 ~ 9.04)	0.528		2.9 (0 ~ Inf)	0.997		11 (1.14 ~ 106.43)	0.038	
Total disability	1.21 (0.65 ~ 2.25)	0.648		0.68 (0.3 ~ 1.54)	0.354		0.89 (0.46 ~ 1.75)	0.743	
Nutritional risk			0.908			0.065			0.194
Yes	1.21 (0.67 ~ 2.19)	0.521		0.9 (0.41 ~ 1.95)	0.37		0.83 (0.45 ~ 1.51)	0.533	
No	1.31 (0.62 ~ 2.77)	0.476		1.12 (0.37 ~ 3.37)	0.038		1.48 (0.7 ~ 3.15)	0.303	
Conscious status			0.320			-			0.286
Awake	1.17 (0.68 ~ 2)	0.57		-	-		0.91 (0.51 ~ 1.62)	0.755	
Confused	1.52 (0.69 ~ 3.37)	0.303		-	-		1.76 (0.74 ~ 4.19)	0.202	
Comatose	4.67 (0.67 ~ 32.36)	0.119		-	-		0.34 (0.05 ~ 2.26)	0.266	
MDR			0.465			0.034			0.760
Yes	1.12 (0.55 ~ 2.27)	0.763		1.16 (0.37 ~ 3.58)	0.339		0.84 (0.39 ~ 1.83)	0.663	
No	1.53 (0.89 ~ 2.62)	0.125		1.65 (1.44 ~ 3.18)	<0.001		1.02 (0.57 ~ 1.84)	0.951	

Subgroup	CPIS score recovery			WBC recovery			NLR recovery		
	OR (95%CI)	*p*-value	*p* for interaction	OR (95%CI)	*p*-value	*p* for interaction	OR (95%CI)	*p*-value	*p* for interaction
Age (years)			0.776			0.053			0.046
< 80	1.28 (0.63 ~ 2.6)	0.499		1.5 (1.27 ~ 1.63)	0.031		0.78 (0.43 ~ 1.4)	0.404	
≥80	1.15 (0.64 ~ 2.1)	0.637		1.59 (1.14 ~ 2.2)	0.069		1.71 (1.44 ~ 2.92)	0.037	
BMI			0.039			0.187			0.344
< 18.5	1.52 (1.09 ~ 2.27)	0.021		0.53 (0.23 ~ 1.2)	0.127		0.74 (0.36 ~ 1.51)	0.41	
18.5 ~ 23.9	1.13 (0.59 ~ 2.14)	0.718		1.01 (0.46 ~ 2.21)	0.978		1.26 (0.67 ~ 2.38)	0.478	
≥24	1.9 (0.45 ~ 8.01)	0.379		2.84 (0.63 ~ 12.86)	0.174		1.88 (0.52 ~ 6.84)	0.337	
ALB (g/L)			0.049			0.042			0.575
< 30	2.61 (1.08 ~ 6.92)	0.043		1.36 (1.15 ~ 3.68)	0.009		1.25 (0.53 ~ 2.95)	0.611	
≥30	0.9 (0.53 ~ 1.53)	0.703		1.08 (0.61 ~ 1.9)	0.142		0.94 (0.56 ~ 1.58)	0.741	
Severity			0.536			0.031			0.457
Mild	1.38 (0.85 ~ 2.22)	0.192		1.91 (1.52 ~ 2.59)	0.022		1.04 (0.65 ~ 1.68)	0.869	
Severe	0.78 (0.12 ~ 5.07)	0.799		1.58 (0.3 ~ 8.4)	0.59		1.72 (0.35 ~ 8.53)	0.508	
Performance status			0.418			0.067			0.235
Self-care	1.11 (0.52 ~ 2.35)	0.79		0.66 (0.23 ~ 1.9)	0.438		1.41 (0.67 ~ 2.96)	0.363	
Semi-disability	4.35 (0.28 ~ 6.49)	0.241		2.54 (0.23 ~ 28.02)	0.447		3 (0.59 ~ 15.36)	0.187	
Total disability	0.94 (0.48 ~ 1.83)	0.85		1.72 (1.38 ~ 2.37)	0.038		0.71 (0.36 ~ 1.4)	0.325	
Nutritional risk			0.295			0.013			0.883
Yes	0.91 (0.51 ~ 1.63)	0.75		1.54 (1.28 ~ 2.06)	0.037		1.2 (0.67 ~ 2.15)	0.533	
No	1.59 (0.75 ~ 3.35)	0.225		1.75 (0.47 ~ 6.51)	0.401		1.09 (0.54 ~ 2.21)	0.811	
Conscious status			0.032			0.028			0.049
Awake	0.99 (0.56 ~ 1.77)	0.876		1.33 (0.72 ~ 2.45)	0.359		1.03 (0.59 ~ 1.79)	0.918	
Confused	1.6 (1.07 ~ 3.66)	0.045		1.35 (1.23 ~ 2.64)	0.019		1.87 (0.75 ~ 4.2)	0.019	
Comatose	1.17 (0.17 ~ 8.09)	0.729		2.57 (0.19 ~ 34.47)	0.476		0.14 (0.01 ~ 1.61)	0.114	
MDR			0.743			0.566			0.036
Yes	1.06 (0.49 ~ 2.29)	0.88		0.75 (0.34 ~ 1.66)	0.481		1.63 (1.36 ~ 2.92)	0.047	
No	1.26 (0.72 ~ 2.2)	0.42		1.12 (0.57 ~ 2.23)	0.739		0.87 (0.5 ~ 1.49)	0.601	

Subgroup	CRP recovery			PCT recovery			PAR recovery		
	OR (95%CI)	*p*-value	*p* for interaction	OR (95%CI)	*p*-value	*p* for interaction	OR (95%CI)	*p*-value	*p* for interaction
Age (years)			0.688			0.044			0.041
< 80	1.63 (1.22 ~ 2.36)	0.006		0.84 (0.38 ~ 1.86)	0.673		1.1 (0.51 ~ 2.37)	0.686	
≥80	1.79 (1.44 ~ 2.63)	<0.001		3.05 (1.54 ~ 6.03)	0.001		1.92 (1.34 ~ 3.67)	0.029	
BMI			0.039			0.560			0.287
< 18.5	0.62 (0.31 ~ 1.26)	0.187		2.43 (1.61 ~ 3.35)	0.041		0.83 (0.34 ~ 2.03)	0.681	
18.5 ~ 23.9	1.73 (1.27 ~ 2.68)	<0.001		2.76 (1.86 ~ 3.62)	0.012		2.09 (1.04 ~ 4.17)	0.037	
≥24	1.42 (0.31 ~ 6.54)	0.654		3.55 (1.56 ~ 22.51)	0.018		1.16 (0.2 ~ 6.88)	0.721	
ALB (g/L)			0.895			0.133			-
< 30	1.53 (1.36 ~ 3.5)	0.008		3.41 (1.16 ~ 10.04)	0.026		-	-	
≥30	1.72 (1.21 ~ 2.04)	<0.001		1.5 (0.84 ~ 2.66)	0.042		-	-	
Severity			0.013			0.593			0.008
Mild	1.28 (1.17 ~ 2.45)	<0.001		1.92 (1.11 ~ 3.31)	0.02		1.9 (1.08 ~ 3.34)	0.025	
Severe	0.77 (0.22 ~ 2.74)	0.312		1.83 (1.24 ~ 6.47)	0.037		0.82 (0.3 ~ 1.23)	0.085	
Performance status			0.032			0.026			0.385
Self-care	0.48 (0.14 ~ 1.63)	0.238		1.29 (0.58 ~ 2.87)	0.529		1.64 (0.69 ~ 3.9)	0.268	
Semi-disability	1.8 (1.34 ~ 9.4)	0.048		1.44 (0.72 ~ 2.8)	0.238		3.01 (0.23 ~ 40.23)	0.404	
Total disability	1.37 (1.18 ~ 2.77)	0.007		2.3 (1.07 ~ 4.9)	0.032		1.03 (0.49 ~ 2.19)	0.274	
Nutritional risk			0.275			0.886			0.017
Yes	1.87 (1.49 ~ 2.55)	0.007		2.74 (1.91 ~ 3.31)	0.039		3.07 (1.4 ~ 6.71)	0.005	
No	1.65 (1.38 ~ 2.91)	0.038		2.25 (1.76 ~ 5.29)	0.026		0.83 (0.41 ~ 1.67)	0.592	
Conscious status			0.063			0.018			0.035
Awake	1.57 (1.01 ~ 1.79)	0.002		2.11 (1.1 ~ 4.05)	0.024		1.19 (0.6 ~ 2.38)	0.615	
Confused	1.62 (1.39 ~ 3.16)	0.034		2.64 (1.04 ~ 6.72)	0.041		2.74 (1.97 ~ 7.77)	0.048	
Comatose	5.33 (0.47 ~ 60.8)	0.178		1.21 (0.12 ~ 4.14)	0.308		1.54 (0.25 ~ 9.66)	0.674	
MDR			0.042			0.039			0.032
Yes	1.12 (0.53 ~ 2.35)	0.211		2.22 (1.87 ~ 5.15)	0.046		2.8 (1.73 ~ 4.41)	0.013	
No	1.28 (0.73 ~ 2.22)	<0.001		1.63 (0.87 ~ 3.06)	0.13		1.64 (1.22 ~ 2.32)	0.072	

Subgroup	D-Dimer recovery			MDR bacteria number reduction			Mortality		
	OR (95%CI)	*p*-value	*p* for interaction	OR (95%CI)	*p*-value	*p* for interaction	OR (95%CI)	*p*-value	*p* for interaction
Age (years)			0.845			0.509			0.798
< 80	0.81 (0.36 ~ 1.82)	0.317		3.35 (1.4 ~ 7.99)	0.006		1.69 (0.41 ~ 6.99)	0.471	
≥80	0.83 (0.44 ~ 1.57)	0.502		2.1 (1.06 ~ 4.18)	0.033		2.17 (0.72 ~ 6.51)	0.168	
BMI			0.171			0.600			0.628
< 18.5	1.09 (0.48 ~ 2.47)	0.831		1.78 (0.87 ~ 3.67)	0.116		1.19 (0.31 ~ 4.58)	0.8	
18.5 ~ 23.9	1.17 (0.57 ~ 2.4)	0.678		1.8 (0.81 ~ 4.01)	0.152		1.37 (0.35 ~ 5.33)	0.65	
≥24	0 (0 ~ Inf)	0.995		0 (0 ~ Inf)	0.997		0.12 (0 ~ Inf)	0.996	
ALB (g/L)			0.069			0.163			0.372
< 30	1.17 (0.57 ~ 2.4)	0.678		2.67 (1.07 ~ 6.67)	0.036		0.61 (0.08 ~ 4.71)	0.633	
≥30	0.83 (0.45 ~ 1.52)	0.543		2.35 (1.19 ~ 4.63)	0.014		2.25 (0.86 ~ 5.88)	0.098	
Severity			0.433			0.088			0.422
Mild	0.87 (0.49 ~ 1.54)	0.625		2.91 (1.63 ~ 5.16)	<0.001		0 (0 ~ Inf)	0.997	
Severe	0.67 (0.12 ~ 3.72)	0.648		1.03 (0.27 ~ 3.94)	0.016		0.73 (0.24 ~ 2.24)	0.586	
Performance status			0.547			0.346			0.043
Self-care	0.49 (0.18 ~ 1.32)	0.157		2.32 (0.38 ~ 14.41)	0.365		0 (0 ~ Inf)	0.999	
Semi-disability	2.98 (0.22 ~ 39.81)	0.409		2.54 (0.23 ~ 28.02)	0.447		0.74 (0.05 ~ 10.55)	0.028	
Total disability	0.88 (0.43 ~ 1.81)	0.733		1.09 (0.59 ~ 2.01)	0.794		1.91 (0.46 ~ 8)	0.294	
Nutritional risk			0.353			0.892			0.207
Yes	0.9 (0.47 ~ 1.71)	0.74		1.95 (1.03 ~ 3.69)	0.04		2.04 (0.65 ~ 6.42)	0.222	
No	0.65 (0.27 ~ 1.54)	0.328		1.57 (0.44 ~ 5.6)	0.484		0.63 (0.11 ~ 3.68)	0.61	
Conscious status			0.280			0.665			0.406
Awake	0.73 (0.38 ~ 1.38)	0.328		2.26 (1.04 ~ 4.92)	0.04		2.28 (0.8 ~ 6.45)	0.122	
Confused	1.24 (0.47 ~ 3.31)	0.664		3.21 (1.24 ~ 8.34)	0.016		0.81 (0.16 ~ 4.15)	0.797	
Comatose	1.17 (0.17 ~ 8.09)	0.876		1.81 (0.15 ~ 22.13)	0.644		2.06 (0 ~ Inf)	0.998	
MDR			0.311			-			0.697
Yes	1.01 (0.46 ~ 2.22)	0.975		-	-		2.68 (0.32 ~ 22.34)	0.363	
No	0.68 (0.34 ~ 1.34)	0.264		-	-		1.66 (0.63 ~ 4.34)	0.302	

OR, odds ratio; CI, confidence interval; BMI, body mass index; ALB, albumin; MDR, multidrug resistant; SpO_2_, peripheral capillary oxygen saturation; CPIS, clinical pulmonary infection score; WBC, white blood cells; NLR, neutrophil-to-lymphocyte ratio; CRP, C-reactive protein; PCT, procalcitonin; PAR, procalcitonin-to-albumin ratio; D-Dimer, fibrin degradation product D.

However, certain subgroups exhibited more pronounced benefits. Specifically, the effects of CHM on hospital stay reduction were more significant in patients aged ≥80 years (*p* for interaction = 0.021), those with BMI < 23.9 (*p* for interaction = 0.008) and MDR-positive infections (*p* for interaction = 0.001), while the reduction in febrile duration was also more pronounced in the BMI < 23.9 subgroup (*p* for interaction = 0.026).

Importantly, for indicators that were not initially associated with CHM treatment in the overall regression analysis, subgroup analyses revealed significant associations in specific populations. Cough and sputum recovery was significantly improved in patients aged ≥80 years (*p* for interaction = 0.030), while SpO_2_ recovery showed a significant association in partially disabled patients (*p* for interaction = 0.046). Improvement in CPIS scores was observed in patients with BMI < 18.5 (*p* for interaction = 0.039), albumin <30 g/L (*p* for interaction = 0.043), and those in confused group (*p* for interaction = 0.032). Additionally, CHM treatment was significantly associated with NLR reduction in patients aged ≥80 years (*p* for interaction = 0.046), those in confused group (*p* for interaction = 0.049), and MDR-positive cases (*p* for interaction = 0.036). PAR levels showed greater improvement in patients aged ≥80 years (*p* for interaction = 0.041), those with mild disease (*p* for interaction = 0.008), at nutritional risk (*p* for interaction = 0.017), partially disabled (*p* for interaction = 0.035) and MDR-positive infections (*p* for interaction = 0.032). Lastly, CHM treatment was significantly associated with reduced mortality in partially disabled patients (*p* for interaction = 0.043).

## Discussion

### Summary of the key findings

This retrospective cohort study evaluated the effects of Chinese herbal medicine (CHM) on the prognosis of older adults with pulmonary infections. Given the complexity of infection management in older adults, including age-related immune decline, comorbidities, and higher risk of multidrug resistance, we selected a comprehensive set of clinical outcomes to capture both short-term recovery and overall disease progression. Our findings demonstrated that CHM treatment was associated with significant clinical benefits, including a reduction in hospital stay duration and febrile period, as well as a higher fever resolution rate compared to conventional treatment. Additionally, CHM-treated patients exhibited greater improvements in inflammatory markers, including WBC, CRP, and PCT, suggesting a potential immunomodulatory effect. Importantly, CHM treatment was also linked to a significant reduction in MDR bacterial counts, highlighting its potential role in infection control. Notably, these core findings were further validated by PSM analysis, 1:1 matching of key baseline confounders balanced all covariates, with CHM’s benefits remaining consistent. This confirms the observed therapeutic value of CHM is not driven by baseline differences, enhancing our conclusions’ robustness. The robustness was further supported by E-value analysis, which demonstrated that unmeasured confounding is unlikely to alter the association between CHM treatment and key clinical outcomes. This complements the PSM and multivariable adjustment, reinforcing that CHM’s beneficial effects are not driven by baseline or unmeasured confounding. Although no statistically significant differences were observed in overall mortality or MDR bacterial reversal, the observed improvements in infection resolution and inflammatory response indicate that CHM may serve as a valuable adjunct to conventional therapy in older adults with pulmonary infections.

### Rationale for the selection of clinical outcomes

The selection of clinical outcomes in this study was guided by their relevance in assessing the severity and prognosis of pulmonary infections. Hospital stay and febrile duration were established key indicators of recovery speed, as prolonged hospitalization and persistent fever are associated with worse prognoses and increased healthcare burdens (19–21). In line with this, a prospective cohort study focusing on elderly patients with community-acquired pulmonary infection has validated that core inflammatory markers including WBC, CRP and PCT are closely correlated with adverse clinical outcomes and can effectively predict the prognosis of this vulnerable population (22). Inflammatory markers (WBC, CRP, and PCT) were included to evaluate systemic immune responses, given their established roles in reflecting infection severity and treatment efficacy (23, 24). Additionally, NLR and PAR were incorporated as composite indicators of inflammation and disease severity. NLR has been linked to worse outcomes in infectious diseases, while PAR integrates inflammatory and nutritional status, making it particularly relevant in older adults (25, 26). The inclusion of these markers allowed for a more nuanced understanding of treatment effects. MDR bacterial count reduction was assessed due to the increasing global threat of antimicrobial resistance, with prior studies suggesting that CHM may have antimicrobial properties and enhance antibiotic efficacy (27). While overall mortality was examined as a secondary outcome, its non-significant difference suggests that short-term hospital-based interventions may have limited effects on long-term survival. Additionally, SpO_2_ and CPIS scores were considered essential for respiratory function assessment, though their improvements were observed primarily in specific subgroups rather than the overall population.

### Pharmacological mechanisms and theoretical basis

Previous studies have explored the potential benefits of CHM in the management of infectious diseases. TCM emphasizes syndrome differentiation over disease diagnosis, formulating treatment principles based on the specific pathological manifestations of the disease at different stages and in different individuals. Older adults with pulmonary infections often present with subtle and atypical symptoms (e.g., absence of typical manifestations such as high fever, cough, or sputum production, but rather non-specific symptoms such as poor appetite, fatigue, and confusion), which poses challenges for early diagnosis and treatment. Through its syndrome differentiation framework, TCM can identify the underlying pathological mechanisms behind these atypical presentations, enabling early intervention even when etiological evidence is unclear or imaging findings are non-specific. This approach helps seize critical therapeutic opportunities and compensates for the timeliness limitations of pathogenetic testing, fully leveraging TCM’s preventive and therapeutic advantage of “preventing disease progression and averting illness before it arises”. As a result, CHM has demonstrated significant efficacy in reducing both hospital length of stay and duration of fever. A study by a research team from Shanghai University of Traditional Chinese Medicine, involving 2,830 COVID-19 patients, confirmed that the integration of CHM with standard care significantly shortened the time to nucleic acid conversion and the hospitalization period, reduced the rate of progression from asymptomatic to mild infection, and markedly decreased the incidence of new-onset symptoms such as fever, cough, and sputum production among asymptomatic carriers (28). Furthermore, a multicenter, double-blind, placebo-controlled, parallel-arm randomized clinical trial by Huang et al. provided additional evidence that CHM intervention effectively shortens the hospital stay of patients with severe pneumonia (29).

### Biological evidence supporting the mechanisms of CHM

Furthermore, patients receiving CHM treatment demonstrated more significant improvements in the levels of inflammatory markers—such as white blood cell count, CRP, and PCT—suggesting that CHM may exert immunomodulatory effects. From a mechanistic perspective, accumulating experimental evidence indicates that CHM formulations modulate key inflammatory signaling cascades involved in pulmonary infection–related immune dysregulation. Twelve antiviral active components were identified in QJHTD, including baicalin, geniposide, and rubescensin, among others. These bioactive compounds have been shown to regulate cytokine networks and attenuate excessive inflammatory responses through inhibition of the JAK2/STAT3 pathway, thereby reducing downstream transcription of pro-inflammatory mediators. This formulation alleviates pulmonary inflammation in animal models by modulating the expression of relevant chemokines and their receptor genes in lung tissue and inhibiting the JAK2/STAT3 signaling pathway (30). Notably, a recent study has confirmed that YQS exerts a potent anti-inflammatory effect in aged mice with pulmonary inflammation by specifically regulating the NF-κB/IL-6 signaling pathway, which directly validates the therapeutic mechanism of YQS in the aged population with pulmonary infection and provides a more targeted experimental basis for our clinical findings (31). Previous studies have also indicated that CHM can mitigate lipopolysaccharide-induced lung injury in mice and exert anti-inflammatory effects by suppressing inflammasome activation. Such regulation of innate immune signaling may help restore immune homeostasis, particularly in older adults characterized by immunosenescence and chronic low-grade inflammation. Further experimental evidence confirms that CHM intervention reduces the abnormally elevated leukocyte count in bronchoalveolar lavage fluid, providing a biological basis for the clinical efficacy of CHM in the treatment of pulmonary infections (32).

The study by Gao et al. (33) demonstrated that a granulated CHM formulation significantly reduced lung viral load in H1N1-infected mice with pneumonia and markedly ameliorated H1N1-induced alterations in peripheral white blood cells through inhibition of the RIG-I/NF-κB/IFN (I/III) signaling pathway. Notably, suppression of NF-κB–mediated transcription represents a convergent anti-inflammatory mechanism shared by multiple CHM-derived flavonoids, which may explain the consistent reductions in CRP and PCT observed in our cohort. CRP, an acute-phase protein produced by the liver in response to inflammation, is not directly targeted by CHM in reducing its levels; rather, the reduction is achieved through holistic multi-target and multi-pathway regulatory mechanisms. For instance, in a diabetic rat model, an aqueous extract of TCM was shown to upregulate the expression of HIF-1α and VEGF, thereby improving local tissue ischemia and hypoxia, which subsequently led to decreased CRP levels (34). Similarly, in an ulcerative colitis model, flavonoid components derived from *Lonicera japonica* (a key herb in Yinqiao San) inhibited the NF-κB signaling pathway, effectively attenuating intestinal barrier damage and gut inflammation, accompanied by a reduction in CRP (35). Regarding procalcitonin (PCT), Gong et al. (36) reported that although the combination of a traditional Chinese medicine injection with carbapenem antibiotics did not reduce the 14-day mortality rate in patients with severe infections, it significantly decreased the levels of inflammatory cytokines—including procalcitonin—in sepsis patients. Liu et al. (37) highlighted that methicillin-resistant *Staphylococcus aureus* (MRSA) can form biofilms, which impede antibiotic penetration and reduce treatment efficacy. Notably, baicalein, a bioactive compound present in QJHTD, was shown to inhibit the accessory gene regulator system in animal models, thereby reducing the expression of staphylococcal enterotoxin A in serum and subsequently lowering both CRP and PCT levels. This anti-biofilm effect of QJHTD has been further verified by an *in vitro* study, which found that QJHTD can directly reduce the load of multidrug-resistant bacteria by inhibiting bacterial biofilm formation, providing a direct mechanistic explanation for the significant reduction in MDR bacterial count observed in the CHM group of our study (38). This anti-biofilm and quorum-sensing inhibitory effect provides a plausible mechanistic link between CHM administration and the observed reduction in MDR bacterial burden in our study. It is particularly noteworthy that the inhibitory effect was more pronounced when baicalein was combined with linezolid, providing a important theoretical basis for combination therapy strategies in older adults with pulmonary infections.

### Implications for clinical practice and future study

CHM treatment has also been associated with a significant reduction in the colonization of MDROs, suggesting its potential value in infection control. Research by Cheng et al. (39) demonstrated that a crude extract of CHM markedly decreased the bacterial load of *Staphylococcus aureus* in lung tissue and effectively alleviated pneumonia-induced inflammatory responses in a model infected by this pathogen. Qin et al. (40) investigated the active constituents within CHM used for treating bacterial pneumonia, with a particular focus on the mechanism of baicalein against multidrug-resistant *Klebsiella pneumoniae*. Their findings suggested that baicalein exerts antimicrobial effects by inhibiting bacterial biofilm formation and disrupting resistance mechanisms, which is consistent with the latest *in vitro* evidence that QJHTD can target MDR bacterial biofilms to reduce bacterial load, further supporting the role of CHM in managing drug-resistant pulmonary infections. Similarly, Xiong et al. (41) and Yang et al. (42) highlighted the role of CHM in modulating immune responses against viral infections, reinforcing the hypothesis that CHM may enhance recovery in pulmonary infections. Furthermore, both YQS and QJHTD contain abundant flavonoids. Song et al. also reviewed the antibacterial modes of herbal flavonoids, emphasizing their role in combating resistant bacteria, and the specific anti-inflammatory mechanism of YQS via the NF-κB/IL-6 pathway in aged animal models further enriches the evidence for flavonoid-rich CHM in the treatment of elderly pulmonary infections, which supports the potential role of CHM in addressing multidrug-resistant infections (37). Although YQS and QJHTD differ in traditional indications and herbal composition, their pharmacological actions converge on shared biological pathways, including modulation of NF-κB– and JAK/STAT–mediated inflammatory signaling, regulation of innate immune responses, and interference with bacterial virulence or biofilm formation. This mechanistic convergence may underlie the consistent clinical improvements observed across different syndrome-based prescriptions in our cohort.

In our study, particular attention was given to older adults with varying degrees of frailty and immunocompromised conditions. Although CHM did not demonstrate significant improvements in overall mortality or the clearance rate of drug-resistant bacteria, stratified analyses revealed substantial benefits in specific patient subgroups. Specifically, patients aged ≥80 years, those with lower body mass index, and those with comorbid drug-resistant bacterial infections exhibited markedly greater reductions in both hospital length of stay and duration of fever. Furthermore, among high-risk subgroups—including individuals with disabilities, nutritional risk, hypoalbuminemia, or altered mental status—CHM treatment resulted in statistically significant improvements in symptom alleviation, oxygen saturation levels, and CPIS. TCM theory emphasizes the concept of the human body as an integrated organic system, prioritizing not only localized pathological changes but also the overall functional state of the body and influences of the external environment during diagnosis and treatment. Older, frail, and immunocompromised patients often present with multiple chronic underlying conditions, such as hypertension, coronary heart disease, diabetes, and renal insufficiency. Conventional anti-infective therapies are often limited in this population due to concerns regarding drug-induced hepatorenal toxicity and polypharmacy-related interactions. In contrast, CHM adopts a holistic regulatory approach, viewing pneumonia and underlying diseases as different manifestations of a unified pathological state. Through syndrome differentiation and targeted treatment, a single herbal formulation can achieve multiple therapeutic effects: addressing both the infectious pathogen and modulating underlying conditions, thereby achieving systemic regulation and avoiding the unintended consequences of fragmented treatment. This approach fully leverages the potential advantages of CHM as a complementary therapeutic strategy. Our findings underscore the importance of adopting individualized treatment strategies for frail older adults, who may derive the greatest benefit from the holistic regulatory effects of CHM. Future research should further investigate the targeted efficacy of specific CHM formulations in vulnerable populations and explore their long-term impacts on functional recovery and quality of life.

In summary, the findings of this study provide strong evidence for the role of CHM as a potential adjunct to conventional therapies in older adults with pulmonary infections. CHM demonstrated clinical benefits, including reduced hospital stay and febrile period, and improved inflammatory markers, suggesting its potential to enhance recovery and infection control. Multivariable adjustment, PSM, and E-value analysis collectively reinforce the stability and credibility of these findings by accounting for measured and unmeasured confounding, supporting CHM’s value as a reproducible adjunctive intervention in this high-risk population. However, the lack of significant changes in mortality and MDR bacterial reversal points to the need for further research to explore long-term effects and optimal treatment regimens. Future studies should focus on validating these findings in larger, multi-center trials, exploring the synergistic effects of CHM with conventional treatments, and investigating the molecular mechanisms behind its immunomodulatory and antimicrobial properties. This approach could offer targeted solutions for frail and immunocompromised older adults, addressing the growing challenge of antimicrobial resistance.

### Limitations

Several limitations should be considered when interpreting our findings. First, as a retrospective cohort study, residual unmeasured confounding cannot be fully ruled out, even with multivariable adjustment, propensity score matching (PSM) and E-value sensitivity analysis. Although E-value analysis verified that our core findings are robust to unmeasured confounding, it cannot eliminate the potential synergistic impact of multiple weak unmeasured factors (e.g., physician treatment preferences, patient adherence to CHM, unrecorded lifestyle habits). Clinicians’ subjective preference for prescribing CHM to more frail or severely ill patients may also introduce selection bias, which may explain the numerical trend of higher mortality in the CHM group. Additionally, the observational design limits the establishment of a definite causal relationship between CHM treatment and clinical outcomes, only an associative one. Moreover, exclusion of patients with incomplete data or severe organ dysfunction may restrict the generalizability of our findings to the broader population of older adults with pulmonary infections. Second, the study was conducted at a single center, which may limit the generalizability of our results to broader populations. Third, variations in CHM prescriptions based on the principle of dialectical treatment introduce heterogeneity, making direct comparisons with standardized Western treatments challenging. This variability also affects the consistency and reproducibility of the observed effects, and though the two CHM formulations applied share core pharmacological mechanisms, such prescriptive heterogeneity may compromise the direct reproducibility of our findings, underscoring the need for future prospective studies with standardized CHM prescriptions. Additionally, while our study assessed MDR bacterial count reduction, the lack of microbiological analysis on the specific antimicrobial effects of CHM prevents a clear understanding of its role in infection control. Further studies are needed to explore the molecular mechanisms and active compounds responsible for its potential antimicrobial properties. Finally, long-term outcomes such as post-discharge survival, functional recovery, and quality of life were not assessed, which limits conclusions about the sustained benefits of CHM. Therefore, the Kaplan–Meier analysis in this study reflects in-hospital survival only and should not be interpreted as an evaluation of long-term mortality outcomes.

## Conclusion

This study provides valuable evidence supporting the use of CHM as an adjunct to conventional treatment for older adults with pulmonary infections. The observed improvements in clinical outcomes suggest that CHM may offer therapeutic benefits in managing pulmonary infections in this vulnerable population. CHM’s benefits were particularly pronounced in high-risk subgroups, including older adults, those with low BMI, patients with disabilities, nutritional risk, low albumin levels, altered consciousness, and those with MDR infections. The role in modulating immune responses and supporting infection resolution underscores its potential as part of an integrated treatment approach. As the global healthcare landscape evolves, incorporating traditional medicine alongside modern medical practices may offer a more comprehensive approach to patient care, particularly for the older adults who often face a greater burden of chronic illness and frailty. Future research should further validate these findings in multi-center prospective studies to enhance generalizability and explore the long-term impacts of CHM in this population.

## Data Availability

Publicly available datasets were analyzed in this study. The datasets generated and/or analyzed during the current study are available from the corresponding author upon reasonable request.

## Footnotes

1
http://www.R-project.org

## Glossary

TCM: Traditional Chinese Medicine
T3SS: Type 3 Secretion System
WHO: World Health Organization
ATS: American Thoracic Society
IDSA: Infectious Diseases Society of America
CHM: Chinese Herbal Medicine
BMI: Body mass index
WBC: White blood cell
NEU: Neutrophil
LYM: Lymphocyte
CRP: C-reactive protein
PCT: Procalcitonin
ALB: Albumin
D-Dimer: Fibrin degradation product D
MDR: Multidrug-resistant
CPIS: Clinical Pulmonary Infection Score
NLR: Neutrophil-to-Lymphocyte Ratio
PAR: Procalcitonin-to-Albumin Ratio
IQR: Interquartile range
CI: Confidence interval
OR: Odds ratios
PSM: Propensity score matching
SMD: Standardized mean difference
AMS: Antimicrobial stewardship
AMR: Antimicrobial resistance
