# Supplementary figures and images for: Chinese herbal medicine as adjunct therapy improves clinical recovery and reduces multidrug-resistant bacterial load in older adults with pulmonary infection: a retrospective cohort study
Source: Front Med (Lausanne). 2026 Apr 28;13:1762339. doi: 10.3389/fmed.2026.1762339 (PMC13160790; doi:10.3389/fmed.2026.1762339)

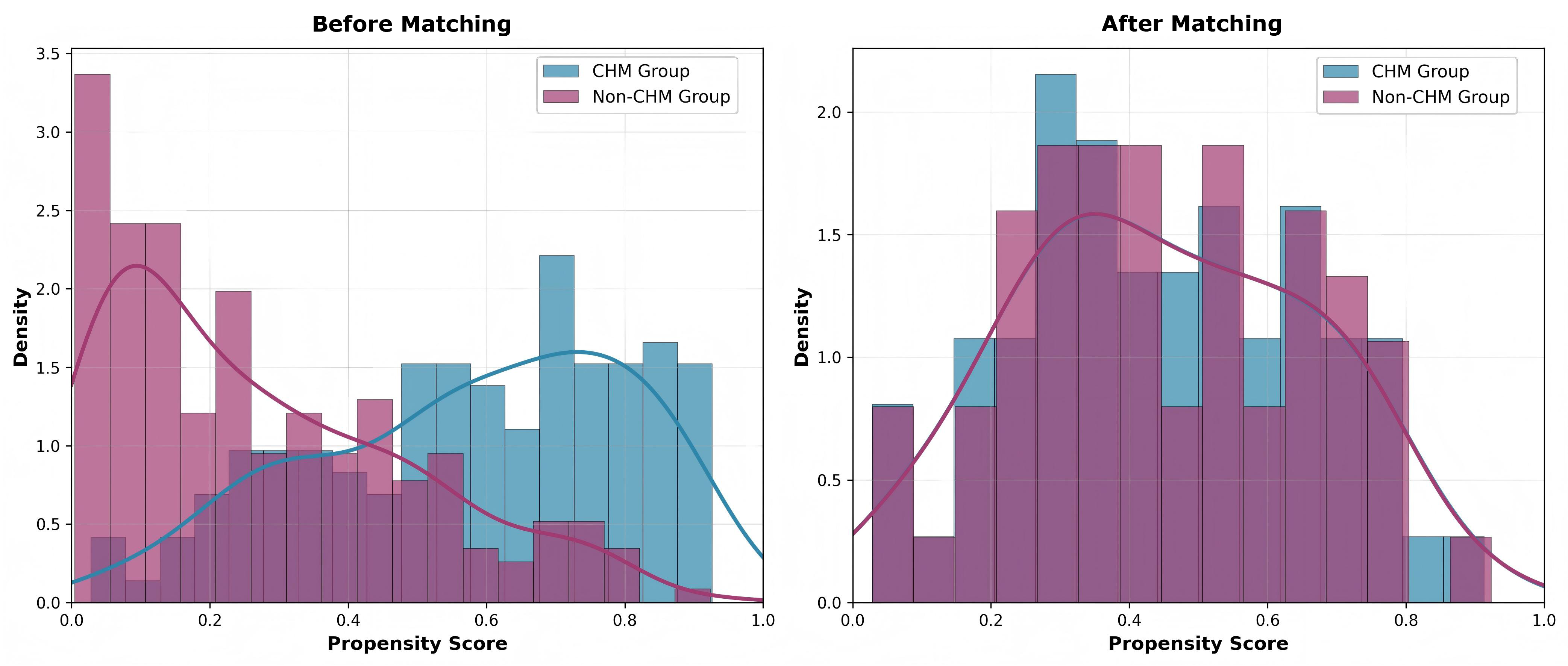

Supplement: Supplementary file 1 [file Image_1.TIF]

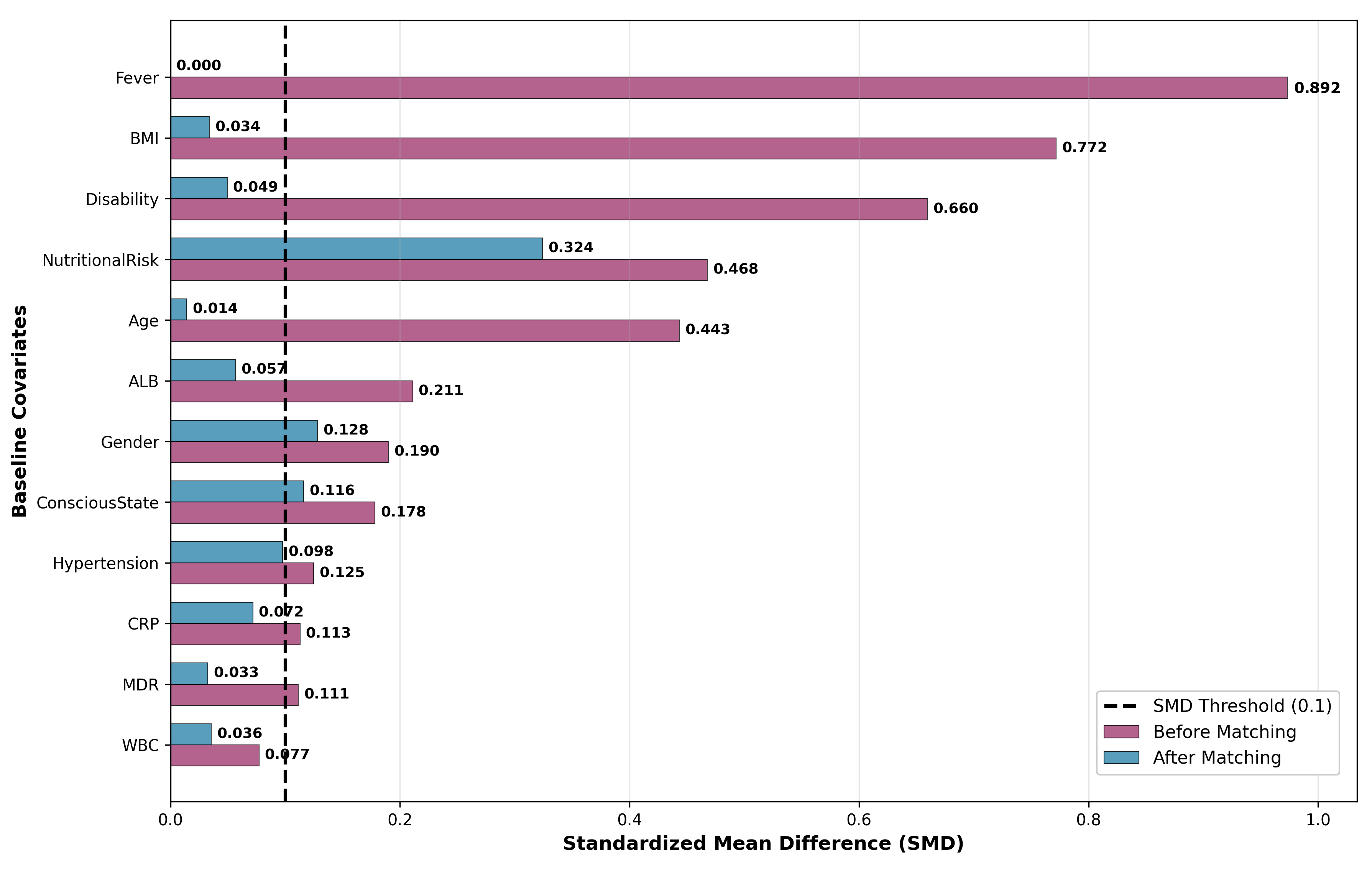

Supplement: Supplementary file 2 [file Image_2.TIF]
