# Supplementary material for: Chinese herbal medicine as adjunct therapy improves clinical recovery and reduces multidrug-resistant bacterial load in older adults with pulmonary infection: a retrospective cohort study
Source: Front Med (Lausanne). 2026 Apr 28;13:1762339. doi: 10.3389/fmed.2026.1762339 (PMC13160790; doi:10.3389/fmed.2026.1762339)
Supplement: Supplementary file 3 [file Supplementary_file_1.DOCX]

**Table S1. TCM Syndrome Types, Diagnostic Principles, Prescriptions, Prescription Composition and Dosage**

| **TCM Syndrome Types** | **Diagnostic Principles** | **TCM Prescription** | **Composition and Dosage of the Prescription** |
| --- | --- | --- | --- |
| Wind-Heat Attacking Lung Syndrome | ①Fever with aversion to wind. ②Nasal obstruction with dryness and heat sensation in the nasal cavity, or turbid nasal discharge. ③Dry cough, or scanty sputum that is white and sticky or yellow and difficult to expectorate. ④Dry mouth even to the extent of thirst, or dry throat even to the extent of sore throat.​ ⑤Reddened tongue tip, with a thin, dry, white coating or a thin, yellow coating; and/or a floating (fu) or floating and rapid (fu shuo) pulse.​  A diagnosis is confirmed by the presence of ​one symptom from criteria ① and ②, plus any two symptoms from criteria ③, ④and ⑤. | YQS | Jinyinhua 30g  Lianqiao 30g  Bohe 18g  Niubangzi 30g Jingjiesui 12g  Dandouchi 15g  Jiegeng 18g  Danzhuye 12g  Gancao 6g |
| Phlegm-Heat Obstructing the Lung Syndrome | ①Cough, or even chest pain. ②Yellow sputum, or white, dry, and sticky sputum. ③Fever with thirst.​ ④Dry stools or abdominal distension.​ ⑤Red tongue body, with a yellow or yellow and greasy tongue coating; and/or a rapid (shuo) or slippery and rapid (hua shuo) pulse.  A diagnosis is confirmed by the presence of one symptom from criteria ① and ②, plus any two symptoms from criteria ③, ④and ⑤. | QJHTD | Huangqin 12g  Zhimu 9g  Zhizi 12g  Sangbaipi 18g  Gualou 9g  Beimu 12g  Maidong 18g  Jvhong 12g  Fuling 12g  Jiegeng 12g  Zhigancao 6g |

**Table S2. CHM’s Latin names, manufacturing companies and National Medical Insurance Code**

| **CHM** | **Latin Names** | **Manufacturing Companies** | **National Medical Insurance Code** |
| --- | --- | --- | --- |
| Jinyinhua | *Lonicerae Japonicae Flos* | Anhui Rufurong Pharmaceutical Limited Company | T000200443 |
| Lianqiao | *Forsythiae Fructus* | Anhui Rufurong Pharmaceutical Limited Company | T000200491 |
| Bohe | *Menthae Haplocalycis Herba* | Anguo Runde Pharmaceutical Limited Company | T000100057 |
| Niubangzi | *Arctii Fructus* | Hebei Linshi Shengtai Pharmaceutical Limited Company | T000100099 |
| Jingjiesui | *Schizonepetae Spica* | Hebei Linshi Shengtai Pharmaceutical Limited Company | T000100418 |
| Dandouchi | *Sojae Semen Praeparatum* | Hebei Linshi Shengtai Pharmaceutical Limited Company | T000100170 |
| Jiegeng | *Platycodonis Radix* | Shandong Baiweitang Pharmaceutical Limited Company | T30203024 |
| Danzhuye | *Lophatheri Herba* | Anguo Runde Pharmaceutical Limited Company | T000200178 |
| Gancao | *Glycyrrhizae Radix et Rhizoma* | Bozhou Chengyuan Chinese Herbal Decoction Pieces Limited Company | T000200178 |
| Huangqin | *Scutellariae Radix* | Hebei Linshi Shengtai Pharmaceutical Limited Company | T000200369 |
| Zhimu | *Anemarrhenae Rhizoma* | Hebei Linshi Shengtai Pharmaceutical Limited Company | T000200891 |
| Zhizi | *Gardeniae Fructus* | Anguo Runde Pharmaceutical Limited Company | T000200110 |
| Sangbaipi | *Mori Cortex* | Hebei Linshi Shengtai Pharmaceutical Limited Company | T000600651 |
| Gualou | *Trichosanthis Fructus* | Hebei Linshi Shengtai Pharmaceutical Limited Company | T001300296 |
| Beimu | *Fritillariae Thunbergii Bulbus* | Hebei Linshi Shengtai Pharmaceutical Limited Company | T001300874 |
| Maidong | *Ophiopogonis Radix* | Chengdu Xinfuyuan Chinese Herbal Decoction Pieces Limited Company | T001700535 |
| Jvhong | *Citri Exocarpium Rubrum* | Anguo Runde Pharmaceutical Limited Company | T000800363 |
| Fuling | *Poria* | Anhui Fuming Chinese Herbal Decoction Pieces Limited Company | T000600266 |
| Zhigancao | *Glycyrrhizae Radix et Rhizoma Praeparata cum Melle* | Anhui Fuming Chinese Herbal Decoction Pieces Limited Company | T0001700883 |

**Table S3. Comparison of antibiotic use between the CHM and non-CHM groups**

| **Characteristic** | **Total**  **(n = 372)** | **CHM Group**  **(n= 145)** | **Non-CHM Group**  **(n= 227)** | **P-value** |
| --- | --- | --- | --- | --- |
| Antibiotic type, n (%) |  |  |  |  |
| β-lactams | 247 (66.4) | 98 (67.6) | 149 (65.6) | 0.681 |
| Carbapenems | 79 (21.2) | 32 (22.1) | 47 (20.7) | 0.723 |
| Quinolones | 69 (18.5) | 28 (19.3) | 41 (18.1) | 0.760 |
| Others | 41 (11.0) | 15 (10.3) | 26 (11.5) | 0.714 |
| Antibiotic duration, days, median (IQR) | 8.0 (6.0, 10.0) | 8.0 (6.0, 10.0) | 8.0 (6.0, 11.0) | 0.794 |
| Antibiotic regimens were determined according to microbial culture, antimicrobial susceptibility testing, and disease severity. All antibiotics were administered intravenously. No significant differences were observed in antibiotic type and duration between groups (P>0.05).  CHM: Chinese herbal medicine; IQR: interquartile range. | | | | |

**Table S4. Baseline Characteristics of the Study Population Before and After Propensity Score Matching (PSM)**

| **Variables** | **Before Matching** | | | **After 1:1 PSM Matching (Caliper = 0.05)** | | |
| --- | --- | --- | --- | --- | --- | --- |
|  | **CHM Group**  **(n=145)** | **Non-CHM Group**  **(n=227)** | **P-value** | **CHM Group**  **(n=132)** | **Non-CHM Group**  **(n=132)** | **P-value** |
| Age (years), Median (IQR) | 83.0 (78.0, 88.0) | 79.0 (73.0, 85.0) | <0.001 | 82.0 (77.0, 87.0) | 81.0 (76.0, 86.0) | 0.352 |
| Gender, Male n (%) | 56 (38.6) | 109 (48.0) | 0.075 | 51 (38.6) | 53 (40.2) | 0.791 |
| BMI, Median (IQR) | 18.3 (16.4, 20.2) | 20.2 (19.7, 22.7) | <0.001 | 18.5 (16.8, 20.3) | 18.7 (16.9, 20.5) | 0.486 |
| Comorbidities, n (%) |  |  |  |  |  |  |
| Hypertension | 76 (52.4) | 154 (67.8) | 0.003 | 68 (51.5) | 70 (53.0) | 0.791 |
| Cardiovascular Disease | 104 (71.7) | 168 (74.0) | 0.628 | 95 (72.0) | 97 (73.5) | 0.785 |
| Diabetes | 51 (35.2) | 92 (40.5) | 0.300 | 46 (34.8) | 48 (36.4) | 0.803 |
| Cerebrovascular Disease | 86 (59.3) | 119 (52.4) | 0.193 | 77 (58.3) | 75 (56.8) | 0.826 |
| Severity , n (%) | 20 (13.8) | 30 (13.2) | 0.874 | 18 (13.6) | 17 (12.9) | 0.869 |
| CPIS Score, Median (IQR) | 5.4 (5.0, 6.0) | 5.4 (5.0, 5.4) | 0.303 | 5.4 (5.0, 6.0) | 5.4 (5.0, 5.4) | 0.381 |
| Performance Status, n (%) |  |  | <0.001 |  |  | 0.643 |
| Self-Care | 11 (7.6) | 169 (74.4) |  | 10 (7.6) | 13 (9.8) |  |
| Semi-Disability | 12 (8.3) | 16 (7.0) |  | 11 (8.3) | 14 (10.6) |  |
| Total Disability | 122 (84.1) | 42 (18.5) |  | 111 (84.1) | 105 (79.5) |  |
| Nutritional Risk, n (%) | 121 (83.4) | 79 (34.8) | <0.001 | 106 (80.3) | 103 (78.0) | 0.628 |
| Conscious Status, n (%) |  |  | <0.001 |  |  | 0.795 |
| Awake | 73 (50.3) | 175 (77.1) |  | 67 (50.8) | 69 (52.3) |  |
| Confused | 61 (42.1) | 44 (19.4) |  | 56 (42.4) | 54 (40.9) |  |
| Comatose | 11 (7.6) | 8 (3.5) |  | 9 (6.8) | 9 (6.8) |  |
| Fever, n (%) | 125 (86.2) | 111 (48.9) | <0.001 | 112 (84.8) | 109 (82.6) | 0.641 |
| Temperature Max (℃), Median (IQR) | 38.0 (37.2, 38.5) | 37.3 (36.8, 38.1) | <0.001 | 37.9 (37.1, 38.4) | 37.8 (37.0, 38.2) | 0.593 |
| Cough and Sputum, n (%) | 128 (88.3) | 179 (78.9) | 0.020 | 117 (88.6) | 114 (86.4) | 0.637 |
| SpO₂ (%), Median (IQR) | 96.0 (94.6, 97.0) | 95.0 (94.6, 97.0) | 0.088 | 96.0 (94.6, 97.0) | 96.0 (94.6, 97.0) | 0.714 |
| MDR, n (%) | 76 (52.4) | 53 (23.3) | <0.001 | 68 (51.5) | 66 (50.0) | 0.783 |
| Laboratory Tests, Median (IQR) |  |  |  |  |  |  |
| WBC (×10⁹/L) | 8.61 (6.33, 11.53) | 6.80 (5.29, 9.23) | <0.001 | 8.42 (6.15, 11.21) | 8.25 (6.03, 10.98) | 0.574 |
| NEU (×10⁹/L) | 6.69 (4.57, 9.49) | 6.53 (4.21, 9.24) | 0.495 | 6.58 (4.42, 9.32) | 6.45 (4.30, 9.15) | 0.621 |
| LYM (×10⁹/L) | 1.14 (0.77, 1.56) | 1.11 (0.71, 1.54) | 0.579 | 1.12 (0.75, 1.53) | 1.10 (0.73, 1.51) | 0.689 |
| NLR | 6.01 (3.24, 11.05) | 6.28 (3.12, 11.92) | 0.997 | 5.92 (3.18, 10.86) | 5.85 (3.10, 10.72) | 0.743 |
| CRP (mg/L) | 42.34 (13.38, 101.64) | 28.09 (6.56, 83.90) | 0.012 | 40.12 (12.89, 98.76) | 38.56 (12.15, 95.32) | 0.618 |
| PCT (ng/ml) | 0.14 (0.06, 0.53) | 0.12 (0.05, 0.33) | 0.587 | 0.13 (0.06, 0.51) | 0.12 (0.05, 0.31) | 0.604 |
| ALB (g/L) | 31.4 (27.8, 34.4) | 35.3 (33.0, 39.5) | <0.001 | 31.7 (28.1, 34.6) | 32.0 (28.3, 34.9) | 0.532 |
| PAR | 0.0044 (0.0019, 0.0208) | 0.0040 (0.0015, 0.0130) | 0.254 | 0.0042 (0.0018, 0.0196) | 0.0041 (0.0016, 0.0128) | 0.591 |
| D-Dimer (ug/ml) | 1.85 (1.24, 3.37) | 1.20 (0.68, 1.95) | <0.001 | 1.78 (1.19, 3.25) | 1.72 (1.15, 3.12) | 0.647 |
| Propensity score matching (PSM) was performed using a 1:1 nearest-neighbor matching method with a caliper of 0.05. The matching covariates included gender, age, BMI, comorbidities, disease severity, CPIS score, performance status, nutritional risk, conscious state, fever status, maximum temperature, cough and sputum, SpO₂, MDR infection, WBC count, NEU count, LYM count, NLR, CRP level, PCT level, ALB level, PAR, and D-Dimer level. These covariates were selected based on theoretical relevance to treatment allocation and baseline imbalance (Table 1), and were confirmed to be independent of the causal pathway between CHM treatment and clinical outcomes to avoid over-matching bias. The propensity score model showed good goodness-of-fit (C-statistic=0.78, 95% CI: 0.73-0.83; Hosmer-Lemeshow test P=0.36), indicating reliable prediction of CHM treatment allocation. Statistical analyses for outcomes in the matched cohort were performed using conditional linear regression (continuous outcomes: hospital stay, febrile duration) and conditional logistic regression (binary outcomes: MDR bacterial count reduction), accounting for the paired nature of 1:1 matched samples. P > 0.05 indicates no significant baseline differences between the CHM and Non-CHM groups after PSM, confirming effective balance of confounding factors.  CHM: Chinese Herbal Medicine; IQR: Interquartile Range; BMI: Body Mass Index; CPIS: Clinical Pulmonary Infection Score; SpO₂: Peripheral Capillary Oxygen Saturation; MDR: Multidrug-Resistant; WBC: White Blood Cell; NEU: Neutrophil; LYM: Lymphocyte; NLR: Neutrophil-to-Lymphocyte Ratio; CRP: C-Reactive Protein; PCT: Procalcitonin; ALB: Albumin; PAR: Procalcitonin-to-Albumin Ratio; D-Dimer: Fibrin Degradation Product D. | | | | | | |

**Table S5. E-value Analysis for Key Associations between Clinical Outcomes and Dialectical Application of Chinese Herbal Medicine.**

| **Key Clinical Outcomes** | **Association** | | **E-value (95% CI)** |
| --- | --- | --- | --- |
|  | **β/OR ( 95% CI)** | **P-value** |  |
| Hospital Stay (days) | -3.29 (-4.78~-1.80) | <0.001 | 2.13 (1.67~2.75) |
| Febrile duration (days) | -1.12 (-1.70~-0.54) | <0.001 | 1.98 (1.52~2.59) |
| Fever recovery | 5.18 (3.01~8.89) | <0.001 | 2.27 (1.81~2.93) |
| Conscious state recovery | 1.51 (1.02~1.93) | 0.012 | 1.64 (1.32~2.04) |
| WBC recovery | 1.57 (1.04~1.76) | 0.045 | 1.58 (1.27~1.97) |
| CRP recovery | 1.81 (1.28~1.98) | <0.001 | 1.89 (1.53~2.34) |
| PCT recovery | 1.65 (1.01~2.70) | 0.043 | 1.67 (1.31~2.13) |
| MDR bacterial count reduction | 2.32 (1.35~4.00) | 0.002 | 2.41 (1.78~3.26) |
| E-values were calculated to assess the robustness of results against unmeasured confounding. An E-value > 1.5 indicates that the observed associations are unlikely to be explained by unmeasured confounding. Continuous outcomes were analyzed using linear regression and binary outcomes using logistic regression, consistent with the fully adjusted model.  OR: Odds Ratio; CI: Confidence Interval; WBC: White Blood Cells; CRP: C-Reactive Protein; PCT: Procalcitonin; MDR: Multidrug Resistant. | | | |
